# Chromosome‐level Asian elephant genome assembly and comparative genomics of long‐lived mammals reveal the common substitutions for cancer resistance

**DOI:** 10.1111/acel.13917

**Published:** 2023-07-03

**Authors:** Xuanjing Li, Pengcheng Wang, Qi Pan, Gaoming Liu, Weiqiang Liu, Olatunde Omotoso, Juan Du, Zihao Li, Yang Yu, Yun Huang, Pingfen Zhu, Meng Li, Xuming Zhou

**Affiliations:** ^1^ CAS Key Laboratory of Animal Ecology and Conservation Biology Institute of Zoology Beijing China; ^2^ University of Chinese Academy of Sciences Beijing China; ^3^ Jiangsu Key Laboratory for Biodiversity and Biotechnology, College of Life Sciences Nanjing Normal University Nanjing China; ^4^ Division of Life Sciences and Medicine University of Science and Technology of China Hefei China

**Keywords:** cancer resistance, comparative genomics, elephants, long‐lived mammals

## Abstract

The naked mole rat (*Heterocephalus glaber*), bats (e.g., genus *Myotis*), and elephants (family Elephantidae) are known as long‐lived mammals and are assumed to be excellent cancer antagonists. However, whether there are common genetic changes underpinning cancer resistance in these long‐lived species is yet to be fully established. Here, we newly generated a high‐quality chromosome‐level Asian elephant (*Elephas maximus*) genome and identified that the expanded gene families in elephants are involved in Ras‐associated and base excision repair pathways. Moreover, we performed comparative genomic analyses of 12 mammals and examined genes with signatures of positive selection in elephants, naked mole rat, and greater horseshoe bat. Residues at positively selected sites of *CDR2L* and *ALDH6A1* in these long‐lived mammals enhanced the inhibition of tumor cell migration compared to those in short‐lived relatives. Overall, our study provides a new genome resource and a preliminary survey of common genetic changes in long‐lived mammals.

## Graphical abstract

A new high‐quality chromosome‐level Asian elephant genome assembly. Expanded gene families in elephants are involved in Ras‐associated and base excision repair pathways. Residues of *CDR2L* and *ALDH6A1* in long‐lived mammals enhanced the inhibition of tumor cell migration.

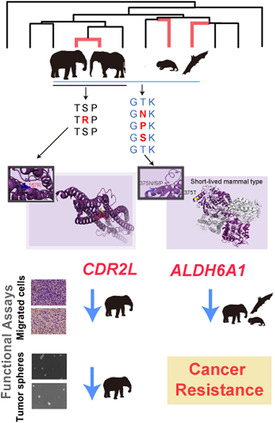

**Abbreviations**
ALDH6A1: aldehyde dehydrogenase 6 family member A1
ALDHs: aldehyde dehydrogenase superfamily
BCC: basal cell carcinoma
bFGF: basic fibroblast growth factor
BUSCO: benchmarking universal single‐copy orthologs
C19orf47: chromosome 19 open reading frame 47
CDR2L: cerebral degenerated‐related protein 2‐like
CDS: coding sequence
COPS3: COP9 signalosome subunit 3
CSF3R: colony‐stimulating factor‐3 receptor
CYLD: cylindromatosis
DMEM: Dulbecco's modified Eagle's medium
DOK2: downstream of tyrosine kinase 2
DOK4: docking protein 4
ECI: early contact inhibition
EGF: epidermal growth factor
FBS: fetal bovine serum
GH: growth hormone
GH/IGF‐1: growth hormone/insulin‐like growth factor 1
GMPR: guanosine monophosphate reductase
GO: gene ontology
Hi‐C: high‐throughput chromosome conformation capture
KEGG: Kyoto Encyclopedia of Genes and Genomes
LIF: leukemia inhibitory factor
LINE: long interspersed nuclear elements
LRIT1: leucine rich repeat, Ig‐like and transmembrane domains 1
LRT: likelihood ratio test
LTR: long terminal repeats
MAPK: mitogen activated protein kinases
MTF1: metal responsive transcription factor 1
MYO6: myosin‐6
MYOT: myotilin
PBS: phosphate buffered saline
PCD: paraneoplastic cerebellar degradation
PSGs: positively selected genes
Rap1: Ras‐associated protein‐1
RBM: RNA‐binding motif
SD: standard deviation
SINE: short interspersed nuclear elements
stLFR: single‐tube long fragment read
TEs: transposable elements
TRAF6: TNF receptor associated factor 6
vHMW‐HA: very high‐molecular weight hyaluronan
WDR7: WD repeat domain 7
XPO1: exportin 1

## 1 INTRODUCTION

Cancer prevention is one of the observed traits in species with extended lifespan. For example, long‐lived mammals, such as the naked mole rat (*Heterocephalus glaber*), bats (e.g., Brandt's bat *Myotis brandtii*), and elephants (family Elephantidae), were suggested to be resistant to cancer (Abegglen et al., 2015; Buffenstein, 2008; Podlutsky et al., 2005). Particularly, the naked mole rat, with an exceptionally maximum lifespan surpassing 37 years, has a low cancer mortality rate (Buffenstein, 2008; Buffenstein & Jarvis, 2002), compared to a similarly‐sized mouse (*Mus musculus*) that only lives 4–5 years (de Magalhães et al., 2005). Previous research revealed several potential cancer resistant mechanisms in naked mole rat. The naked mole rat fibroblasts exhibit a remarkable hypersensitivity to contact inhibition, also referred to as early contact inhibition (ECI) (Seluanov et al., 2009). In addition, naked mole rat tissues can secret very high‐molecular weight hyaluronan (vHMW‐HA), which is not only believed to be an adaptive response to subterranean habitat, but also serves as anti‐cancer strategy (Tian et al., 2013) because vHMW‐HA represses tumor angiogenesis, promotes apoptosis, and inhibits inflammatory responses (Liu et al., 2008; Zhao et al., 2019). The Brandt's bats were recaptured after 41 years and were considered the longest‐lived mammals when adjusted for body size (Podlutsky et al., 2005). Low incidence of cancer in bats, especially in the *Myotis*, might involve bat‐specific regulatory activities of microRNAs that function tumorigenesis pathways (Huang et al., 2016, 2019), downregulation of the growth hormone/insulin‐like growth factor 1 (GH/IGF‐1) pathway, unique sequence change in the GH‐receptor (Seim et al., 2013), repression of telomerase activities (Gomes et al., 2011), and positive selection in telomere‐associated genes (Huang et al., 2019; Morgan et al., 2013). These findings received increased attention as they provide targets for clinical intervention employed in humans and mice (Guevara‐Aguirre et al., 2011; Ikeno et al., 2009).

If an increase in cell division corresponds with an increased risk of cancer, then one would expect elephants to have higher cancer incidents compared to humans (Peto et al., 1975). Despite having ~1000 times more cells than humans, elephants have a cancer mortality rate of <5%, compared to humans with between 11 and 25% cancer mortality rate (Abegglen et al., 2015). The maximum lifespan of the African bush elephant (*Loxodonta africana*) is estimated at 65 years, while that of the Asian elephant (*Elephas maximus*) is ~80 years (de Magalhães et al., 2005). Elephant genome includes possession of extra copies of TP53 gene, a known tumor suppressor gene with roles in DNA damage response, apoptosis, and cell cycle, which may result in the lower cell death response to cellular stresses (Abegglen et al., 2015; Sulak et al., 2016; Tollis et al., 2021). In addition, *p53* is tightly regulated by the *Mdm2* (murine double minute‐2) and their interaction generates a variety of BOX‐I MDM2 binding motifs in 20 copies of TP53 isoforms, which would enhance sensitivity to DNA damage and suppress tumor (Padariya et al., 2022). In addition to extensive sequence conservation at TP53 retrogene loci found in elephants (Tollis et al., 2021), elephants also have expanded copies of the leukemia inhibitory factor (LIF) gene, a downstream target of TP53 that induces apoptosis (Vazquez et al., 2018). These observations support the initiation of using elephant as model to investigate how evolution controls tumor in mammals.

Convergent phenotypes among unrelated taxa are commonly subjected to similar evolutionary pressure. Tumor suppression, though it evolutionary correlated with large body size and long lifespan across animals (Caulin & Maley, 2011; Gorbunova & Seluanov, 2009), the underlined genetic changes seems to have independently evolved across distant mammalian taxa (Omotoso et al., 2021). Here, using the high‐quality genome resource of several long‐lived mammals, we aimed to explore the “convergent” or common substitutions in long‐lived mammals. To this end, we first generate a chromosome‐level genome assembly of Asian elephants and then employed comparative genomic analysis of 12 mammalian genomes to identify positively selected genes in long‐lived mammals. Our comparative analysis showed the signal of positive selection on *ALDH6A1* and on *CDR2L*. We further performed experimental assays to show that residues at the positively selected sites in *ALDH6A1* and *CDR2L* suppress the migration of tumor cells. To sum up, our results provide a new high‐quality genome resource for elephant and partially indicate that there is common substitutions response to cancer resistance in long‐lived mammals. These genomic resources and discoveries could be beneficial for the future researches in identifying effective cancer therapeutic approaches.

## 2 MATERIALS AND METHODS

### 2.1 Sampling and genome sequencing

The fresh blood sample of a male Asian elephant was gifted by wild elephant valley, Xishuangbanna, Yunnan Province, China. The sample was kept with anticoagulant at −80°C at the Institute of Zoology, Chinese Academy of Sciences. All collection and processing of blood samples were conducted in accordance with the guidelines of Institutional Animal Care and Use Committee of the Institute of Zoology, Chinese Academy of Sciences. DNA was extracted with phenol‐chloroform method and its quality was evaluated with agar gelatin electrophoresis. After obtaining high‐molecular‐weight DNA, the single‐tube long fragment read (stLFR) library (Wang et al., 2019) was constructed and then sequenced with a paired‐end 100 bp sequencing strategy on BGISEQ‐500 high‐throughput sequencing platform. To generate a chromosomal‐level assembly of the Asian elephant genome, a Hi‐C (high‐throughput chromosome conformation capture) library (Burton et al., 2013) was further constructed and sequenced with BGISEQ‐500 platform. To evaluate the quality of the assembly, we collected an additional placenta sample from a female Asian elephant in Xishuangbanna and used the same method described above to isolate DNA. The fragment library (insert length was 250 bp) of this sample was constructed and sequenced with a paired‐end 150 bp sequencing strategy on BGISEQ‐500.

To facilitate annotation of the Asian elephant genome, total RNA from the placenta sample was isolated and the fragment library (insert length was 250 bp) was built based on the Iso‐Seq protocol. The fragment library of RNA was sequenced on the BGISEQ‐500 platform with a paired‐end 150 bp sequencing strategy and aimed at 6 Gb data. The full‐length transcripts were sequenced on Pacbio Sequel platform at BGI and aimed at about 30 Gb data.

### 2.2 Genome assembly and evaluation

We employed the following criteria to filter the raw reads from the stLFR library: (1) the reads that have more than 50% bases with a quality of less than five; (2) the reads that have adapters; and (3) the reads that have more than 5% N bases. After filtering, we employed Supernova (Weisenfeld et al., 2017) to assemble the genome and then used Gapcloser (Luo et al., 2012) to close the gaps. The completeness was assessed using Benchmarking Universal Single‐Copy Orthologs (BUSCO) (Simão et al., 2015).

The Hi‐C data were used for generating a chromosomal‐level assembly. HiC‐Pro v 2.8.0 (Servant et al., 2015) and bowtie2 v. 2.2.5 (Langmead et al., 2009) were used to filter the raw Hi‐C data. Then, Juicer v. 1.5 (Durand et al., 2016) and 3D de novo assembly v. 170123 (Dudchenko et al., 2017) were employed to assign scaffolds to 28 pseudochromosomes.

To evaluate the accuracy and quality of the current version of Asian elephant genome assembly, reads from the fragment library in the Trim Galore v0.4.2 (http://www.bioinformatics.babraham.ac.uk/projects/trim_galore/) were filtered with default parameters, and then, the clean reads were aligned to the new assembly with BWA MEM (Li & Durbin, 2009). Samtools (Li et al., 2009) was employed to measure the mapping rate and the coverage.

### 2.3 Genome annotation

We combined de novo and homology methods to identify the tandem repeats and interspersed repeats in the Asian elephant genome. We used RepeatModeler v. 2.0.1 (https://github.com/Dfam‐consortium/RepeatModeler) with RepeatMasker v. 4.1.2 (Chen, 2004), RECON v. 1.08 (Bao & Eddy, 2002), RepeatScout v. 1.0.6 (Price et al., 2005), TRF v. 4.0.9 (Benson, 1999), and RMBlast v. 2.11.0 (Johnson et al., 2008) to predict the repetitive sequences. The LTR_Finder (Xu & Wang, 2007) was used to find full‐length LTR retrotransposons in the genome. The RepeatMasker v. 4.1.2 (Chen, 2004) and repeat database, and Dfam v. 3.2 (Bao et al., 2015) were used to identify the repetitive sequences based on homology sequences. All predicted repetitive sequences were combined, and then, the short fragments (<20 bp) and the overlapping repeats were removed.

To predict the gene structure in the Asian elephant genome, we combined de novo prediction, homology‐based prediction, and transcriptome‐based methods to analyze the genome. The AUGUSTUS (Stanke et al., 2006) was used to predict the gene structure. The protein sequences of cattle (*Bos taurus*), African bush elephant (*Loxodonta Africana*), rock hyrax (*Procavia capensis*), aardvark (*Orycteropus afer*), and Florida manatee (*Trichechus manatus*) were used as homology sequences in Genewise (Birney et al., 2004) to predict gene structure. The protein sequences of aardvark (accession numbers PRJNA237355) and Florida manatee (accession numbers: PRJNA189960) were downloaded from the National Center for Biotechnology Information (https://www.ncbi.nlm.nih.gov/), while sequences of other species were obtained from the Ensembl release 99 (http://www.ensembl.org/).

The SMRTanalysis v. 6.0.0 (https://www.pacb.com/support/software‐downloads/) was used to produce the consensus transcripts from the iso‐seq sequencing data. The reads from the fragment library were used to correct the consensus transcripts. The high‐quality consensus transcripts were used as evidence to predict gene structure in Trinity v. 2.12.0 (Grabherr et al., 2011). The Glean v. 1.0.1 (Elsik et al., 2007) was used to combine the results from the above three methods and filter the results. Predicted genes were filtered out if (1) there is only one de novo evidence to support the gene structure; (2) the length of CDS is less than 150 bp; and (3) the overlap length ratio with transposable elements (TEs) is less than 0.2. The mammalia_odb9 database from BUSCO was used to evaluate the quality of the gene set, and whole‐genome synteny with cattle and human genome, k‐mer analysis were used to estimate the genome quality. The database from SwissProt (Boeckmann et al., 2003), TrEMBL (Boeckmann et al., 2003), KEGG (Kanehisa & Goto, 2000), InterPro (Mitchell et al., 2019), and GO (Ashburner et al., 2000) were used to annotate the gene function. We also identified noncoding RNAs of the Asian elephant genome. The tRNAscan‐SE (Lowe & Eddy, 1997) was used to identify the tRNA, and the RNAmmer (Lagesen et al., 2007) was used to predict rRNA. The INFERNAL (Nawrocki & Eddy, 2013) and Rfam v. 14.5 (Griffiths‐Jones et al., 2005) were used to predict miRNA and snRNA.

### 2.4 Gene family clustering

Homologs among the following 12 mammals: Asian elephant (*Elephas maximus*), African bush elephant (*Loxodonta africana*), cattle (*Bos taurus*), dog (*Canis familiaris*), small Madagascar hedgehog (*Echinops telfairi*), naked mole rat (*Heterocephalus glaber*), human (*Homo sapiens*), gray short‐tailed opossum (*Monodelphis domestica*), aardvark (*Orycteropus afer*), platypus (*Ornithorhynchus anatinus*), cape rock hyrax (*Procavia capensis*), and greater horseshoe bat (*Rhinolophus ferrumequinum*) were estimated. The greater horseshoe bat rather than Brandt's bat (*Myotis brandtii*) was selected because the greater horseshoe bat is also long‐lived bats (with longevity estimated to be 30.5 years) and its genome assembly is chromosome level (Rhie et al., 2021). We extracted the longest transcripts of each gene and formatted the corresponding protein sequences from all 12 mammals into a Blastp database (Johnson et al., 2008). We further employed the protein sequence alignment of each species and confirm the homologous sequences in Blastp database with the e‐value was 1 × 10^−7^. Using the protein sequences alignment, OrthoMCL (Li et al., 2003) was used to cluster orthologous protein sequences. The sequence alignment was performed for each ortholog in the MUSCLE program (Edgar, 2004), and the alignment was filtered using Gblocks v. 0.91b (Castresana, 2000).

Gene family expansions and contractions were analyzed using CAFÉ program (De Bie et al., 2006). The number of expanded and contracted genes for each branch and node of the phylogenetic tree was estimated and the significantly “expanded and contracted gene families” are identified when an exact *p* value (Viterbi method) ≤0.01. Significantly overrepresented GO (Gene Ontology) terms were identified using the topGO (Alexa & Rahnenführer, 2009) package in R (https://www.r‐project.org/), and the Benjamini and Hochberg FDR correction was applied. Significantly overrepresented GO terms were identified with corrected *p* values of ≤0.05.

### 2.5 Identification of positively selected genes

In order to measure selective pressures acting on protein‐coding genes in long‐lived mammals, we screened the signature of positive selection of all orthologs using the CodeML program implemented in PAML package v. 4.8 (Yang, 2007). The optimized branch‐site model (Yang & Dos Reis, 2010) was used to detect signatures of positive selection along specific lineages. Several recent studies have called into question any evidence from codon‐based models of sequence evolution that do not take into account simultaneous double mutations or variation in silent substitution rates (Venkat et al., 2018; Wisotsky et al., 2020). The Busted model in the Hyphy package was also used to confirm the selection signals of each ortholog on each lineage (Kosakovsky Pond et al., 2019). In this analysis, two groups of foreground branches were set: elephant lineage (Asian elephant + African bush elephant) and long‐lived mammals (Asian elephant + African bush elephant + naked mole rat + greater horseshoe bat). The latter group was formulated due to these four mammals have been recognized to show the strong ability of anti‐cancer and long‐lived. Finally, the phylogenetic tree of 12 mammals was retrieved from TimeTree (http://www.timetree.org/) (Kumar et al., 2017).

Gene Ontology and Kyoto Encyclopedia of Genes and Genomes (KEGG) pathway enrichment analyses of these positively selected genes were implemented in clusterProfiler (Yu et al., 2012), with the following parameters, OrgDb = org.Hs.eg.db, fun = “enrichGO,” ont = “BP” (“MF” and “CC”), pvalueCutoff = 0.05, and qvalueCutoff = 0.2. The functional categories with *p* value less than 0.05 were considered to be statistically significant.

### 2.6 Plasmid construction

To explore the roles of residues at positively selected sites in cancer resistance, we constructed several plasmids for functional cellular assays. Complete *ALDH6A1* coding sequence of elephants, greater horseshoe bat, naked mole rat (*ALDH6A1* ^*AAC*^, *ALDH6A1* ^*AGT*^, and *ALDH6A1* ^*CCG*^), and mouse (*ALDH6A1* ^*ACA*^) was used as wild type. In addition, the positively selected site of *ALDH6A1* was mutated, with codons in long‐lived mammals mutated to mouse genotype (*ALDH6A1* ^*AACACA*^, *ALDH6A1* ^*AGTACA*^, *ALDH6A1* ^*CCGACA*^) and codon in mouse was mutated to long‐lived mammals genotype (*ALDH6A1* ^*ACAAAC*^, *ALDH6A1* ^*ACAAGT*^, *ALDH6A1* ^*ACACCG*^). Similarly, the complete *CDR2L* coding sequence of Asian elephant (*CDR2L* ^*AGA*^) and mouse (*CDR2L* ^*AGT*^) was used as wild type, and the positively selected site was mutated correspondingly (*CDR2L* ^*AGAAGT*^ and *CDR2L* ^*AGTAGA*^). The wild and mutant sequences were synthesized by Beijing Genomics Institute (BGI) and cloned into pEGFP‐N1 vectors (Clonetech) separately.

### 2.7 Cell culture and transfection

Dulbecco's modified Eagle's medium (DMEM, Gibco; Thermo Fisher Scientific, Inc.) with 10% fetal bovine serum (FBS, Gibco; Thermo Fisher Scientific, Inc.) and 1% penicillin–streptomycin were used to cultivate A549 cells (which is a widely used human lung adenocarcinoma cell line) and then cultured in an incubator containing 5% CO_2_ at 37°C. The fresh culture medium was changed daily, and the logarithmic growth cells were collected for further experiments.

Cells were plated in 24‐well plates, at 70% confluence; we performed cell transfection using Lipofectamine™ 3000 kit following the manufacturer's protocol. At 24 h post‐transfection, cells were prepared for transwell migration assay.

### 2.8 Transwell migration assay

In the migration experiment, A549 cells were diluted to 1 × 10^5^/mL with serum‐free medium and seeded into the upper chamber of a transwell insert (8.0 μm pore size; CORNING Inc.), and a medium with 10% FBS was added to the lower chamber as a chemoattractant to induce A549 cell migration. After incubation at 37°C, 5% CO_2_ for 48 h, the transwell chamber was taken out and the medium in the well was discarded and washed with PBS twice. The cells were then fixed in methanol for 30 min and stained with crystal violet (Beyotime) for 15 min. After staining, the upper immobile cells were slightly wiped off with a cotton swab, observed, and photographed by the microscope. The number of migrated cells was computed with ImageJ software.

### 2.9 Tumor sphere formation assay

The validated CDR2L/ALDH6A1 and their mutant plasmids were co‐transfected with pMD2.G and psPAX2 into 293FT cells, and the supernatant was filtered after 2 days of culture. Then, A549 cells were infected with the supernatant and screened with puromycin. Stably expressed cells were selected for subsequent tumor sphere formation assay. For assessing the sphere‐forming ability, 1 × 10^3^ cells were seeded in six‐well ultra‐low attachment plates (Corning) in serum‐free medium containing DMEM (Gibco; Thermo Fisher Scientific, Inc.) supplemented with 20 ng/mL basic fibroblast growth factor (bFGF; HARVEYBIO), 20 ng/mL epidermal growth factor (EGF; Invitrogen), and B27 supplement (Invitrogen). Sphere size and number were measured after 7 days of seeding. Images and numbers of tumor spheres were taken and counted with the use of KEYENCE BZ‐X800LE microscope (KEYENCE, Osaka, Japan). Tumor spheres greater than 75 μm were counted.

### 2.10 Statistical analysis

After collecting the number of migration cells, statistical analyses were performed in GraphPad Prism software version 8.0 for Windows (GraphPad Prism Software, San Diego, CA, USA). The data were expressed as the means ± standard deviation (SD). Statistical significance between the two groups was estimated by Student's t–test. Differences with *p* value <0.05 were considered to be statically significant.

## 3 RESULTS

### 3.1 Sequencing, assembly and annotation of the Asian elephant genome

**FIGURE 1:**
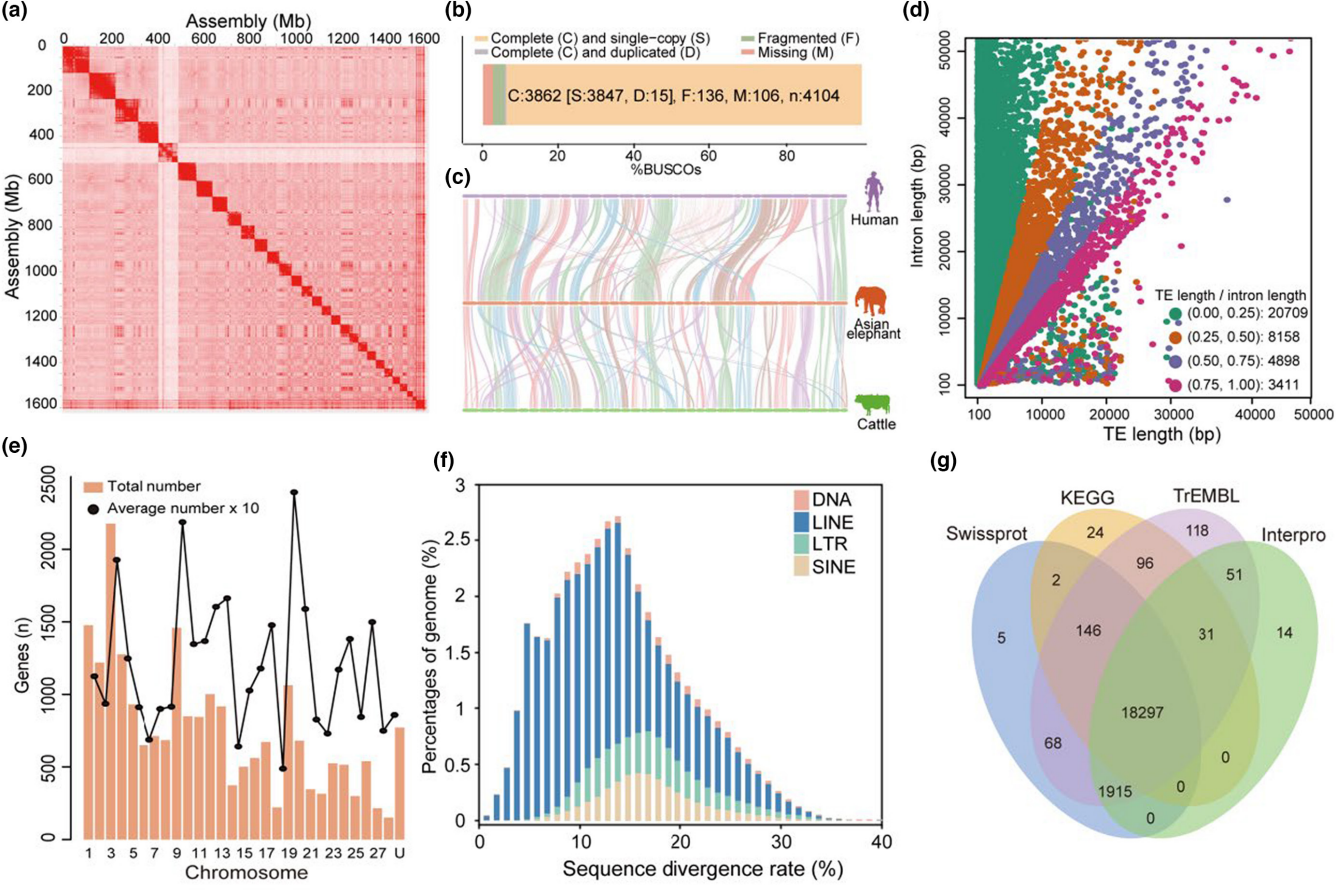
Asian elephant genome assembly and annotation. (a) Genome‐wide heatmap of Hi‐C interactions among 28 chromosomes of Asian elephant. (b) Quality evaluation of the assembled genome in BUSCO software with “mammalia_odb9” dataset. (c) Chromosome synteny among cattle, human, and Asian elephant. The transposon elements (TE) and predicted genes of the Asian elephant genome: (d) Relationship between the intron length and TE length. (e) The number of predicted genes in each chromosome and solid lines denote 10 times of the average number of genes in each numbered chromosome along 10 Mb windows with a step size was 2 Mb. (f) Divergence distribution of repeat elements in the Asian elephant genome. (g) The Venn diagram of functional annotation in the predicted gene set using four protein databases (Swissprot, KEGG, TrEMBL, and Interpro).

A total of 2525.21 million (78.18×) stLFR clean reads were obtained for genome assembly. K‐mer analysis (*k* = 17) estimated size of 3.54 Gb genome (Figure S1, Table S1). In general, the average GC contents of the Asian elephant were similar to other mammals and the GC contents are ~40% (Figure S2). Then, Hi‐C sequencing data were used to anchor the scaffolds and contigs into chromosomes, which yielded 733.58 million valid Hi‐C pair reads with ~97.12% of the bases successfully aligned to 28 chromosomes (2*n* = 56). Approximately 98.21% of reads from the fragment library could be aligned to the new assembly genome (Figure 1a, Table 1, Figure S3, Tables S2–S4). BUSCO analysis of genome assembly showed high BUSCO scores (94.1%) (Figure 1b, Table S5), reflecting that the majority of the assemblies were with high quality and continuity (Figure 1c). The new Asian elephant genome assembly shows a 20‐fold increase of scaffold N50 length (Table 2) compared to with previous Asian elephant genome assembly generated using short reads (Tollis et al., 2021). Moreover, gaps in the new genome have been largely reduced and most evaluation indexes of genome quality show the new genome assembly is of better quality (Table 2).

**TABLE 1:** Statistics of stLFR and Hi‐C sequencing for the Asian elephant genome assembly.

Features	stLFR		Hi‐C	
	Contigs	Scaffolds	Contigs	Scaffolds
Total number	89,150	58,642	89,150	57,037
Total length	3,132,914,974	3,226,711,689	3,132,914,974	3,227,514,189
Gap N	/	93,796,715	/	94,599,215
Average length	35,142.06	55,023.91	/	/
N50	200,987	40,156,848	200,987	118,153,099
N90	45,879	4,887,711	45,879	73,467,117
Maximum length	1,518,750	163,308,399	1,518,750	237,448,774
Minimum length	48	102	48	102
GC content	40.82	40.82	/	/

**TABLE 2:** Summary of Asian elephant genome assembly generated in this study compared with existing Asian elephant genome assembly (Tollis et al., 2021).

Features	Our genome data		Tollis et al. (2021)	
	Contigs	Scaffolds	Contigs	Scaffolds
Assembly length	3.13 Gb	3.23 Gb	2.98 Gb	3.13 Gb
Longest	1.52 Mb	163.31 Mb	731 kb	14.6 Mb
Number	89,150	58,642	90,662	6954
N50	201.0 kb	40.16 Mb	79.8 kb	2.77 Mb
L50	4602	24	10,736	336
Percent genome in gaps	0.00	2.90	0.09	4.88
BUSCO results	C: 93.7% [D: 0.4%] F: 3.3%, M: 2.6%, *n*: 4104		C: 91.5% [D: 0.4%] F: 5.7%, M: 2.8%, *n*: 4104	

Abbreviations: BUSCO, Benchmarking Universal Single Copy Orthologs; C, complete; D, duplicated; F, fragmented; M, missing.

Repetitive sequences account for 68.05% of the Asian elephant genome (Table S6), with DNA transposon, long interspersed nuclear elements (LINE), short interspersed nuclear elements (SINE), and long terminal repeats (LTR) in the genome was 1.29%, 47.59%, 4.54%, and 11.38%, respectively (Figure 1d, Table S7). Besides, the number of miRNA, rRNA, snRNA, and tRNA is 2429, 312, 1284, and 21,499 respectively (Table S8). A total of 21,955 genes, of which 96.48% on the 28 assembled chromosomes were predicted (Figure 1e–g). In addition, approximately 93.07%, 84.70%, 94.38%, and 92.50% of protein‐coding genes could be assigned functions based on Swissprot, KEGG, TrEMBl, and Interpro databases, respectively (Tables S9 and S10).

### 3.2 Gene family evolution

**FIGURE 2:**
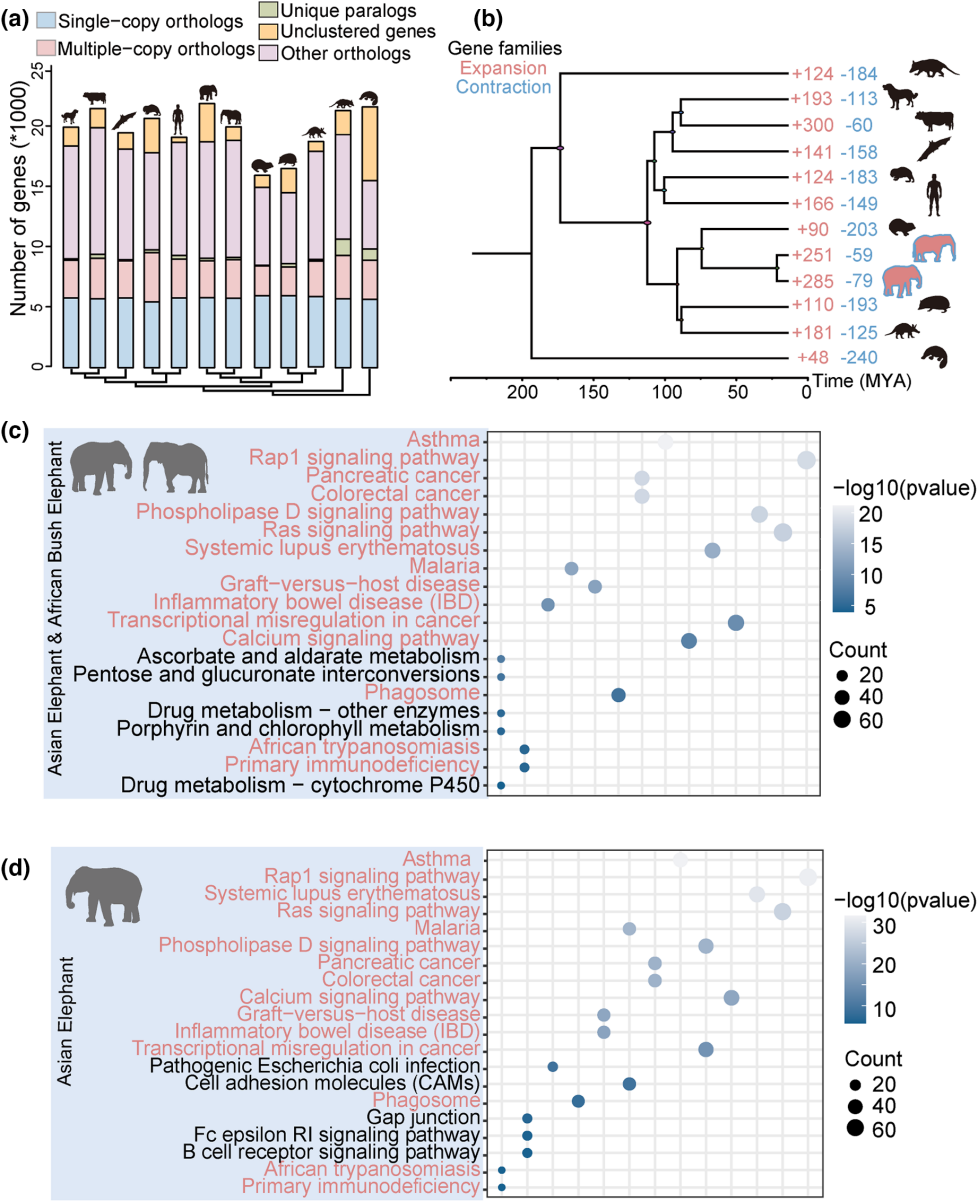
Gene family evolution and enrichment of elephants. (a) The number of homolog genes in the 12 mammals analyzed in this study. (b) Phylogenetic tree and divergence time estimated for the Asian elephant and other mammals. The number of significantly expanded (red) and contracted (blue) gene families is designated on each branch. Top 20 KEGG pathways most significantly enriched with expanded gene families in Asian elephant and African bush elephant (c) and Asian elephant only (d).

We identified 318 significantly expanded and one contracted gene families in the ancestral branch leading to Asian elephant and Africa bush elephant (Figure 2a,b, Tables S11 and S12). Expanded families enriched in signaling pathways, such as Ras‐associated protein‐1 (Rap1) signaling pathway (corrected *p* value = 3.84 × 10^−18^), Phospholipase D signaling pathway (corrected *p* value = 9.91 × 10^−18^), calcium signaling pathway (corrected *p* value = 2.93 × 10^−8^), pentose and glucuronate interconversions pathway (corrected *p* value = 1.01 × 10^−6^), and B‐cell receptor signaling pathway (corrected *p* value = 4.30 × 10^−3^) (Figure 2c, Figure S5a, Tables S13–S15). *Rap1* is an important regulator of cellular migration and polarization as a small GTPase in the Ras‐related protein family. Previous studies have suggested that *Rap1* has an important role in tumor development (Alemayehu et al., 2013; Lin et al., 2015; Maxson et al., 2013; Yang et al., 2015) and tumorigenesis (Shah et al., 2018; Yang et al., 2017). Active Rap1 inhibits tumor invasion and metastasis in bladder, lung, and brain (Lyle et al., 2008; Vallés et al., 2004).

A total of 251 significantly expanded and 59 significantly contracted gene families were identified in Asian elephant (Figure 2b, Table S12). Expanded gene families in Asian elephant lineage were mostly enriched in similar categories that enriched by the expanded families in the ancestral lineages of elephants (Figure 2d, Figure S5b, Tables S16 and S17). However, there are some unique enriched pathways in Asian elephant (Table S17), for example, base excision repair pathway (corrected *p* value = 3.10 × 10^−4^), apoptosis pathway (corrected *p* value = 2.30 × 10^−3^), and longevity regulating pathway (corrected *p* value = 9.00 × 10^−3^). In addition, we found functional KDM4 family duplicated in Asian elephant lineage (Table S18). The KDM4 family is one of KDMs subfamily and plays important roles in many different processes, including regulation of gene transcription, epigenetic silencing, and DNA repair (Kim et al., 2012). More so, irregulating of KDM4 protein can increase the risk of oncogenesis (Chi et al., 2010; Cloos et al., 2008).

### 3.3 Positively selected genes in elephants

Of 4444 single‐copy orthologous from 12 mammals, we identified 618 positively selected genes (PSGs) in the ancestral lineage leading to Asian elephant and African bush elephant (Table S19). Eight PSGs (*DOK4*, *C19orf47*, *TRAF6*, *XPO1*, *WDR7*, *DOK2*, *CYLD*, and *LRIT1*) involved in mitogen activated protein kinases (MAPK) signaling pathway (*p* value = 0.04) (Figure S6a,b, Table S20). Interestingly, MAPKs are activated in response to a variety of stimuli like UV damage and oxidative stress (Chang & Karin, 2001; Corre et al., 2017). In addition, p38MAPK activates cellular responses by regulating various targets, including the prototypical tumor suppressor p53 (Bulavin et al., 1999; Bykov et al., 2018; Hager & Gu, 2014; Mello & Attardi, 2018). Furthermore, 16 genes were enriched in Epstein–Barr virus infection (*p* value = 0.02), of which is the first human tumor virus (Gujer et al., 2015). Other PSGs are also of interesting; for instance, Myosin‐6 (*MYO6*, likelihood ratio test (LRT) *p* value = 6.67 × 10^−11^) is significantly upregulated in prostate and breast cancer (Duan et al., 2020; Zhang et al., 2016). Tyrosine kinase 2 (*DOK2*, LRT *p* value <0.01) is a well‐known tumor suppressor gene (Pei et al., 2021) and regulated hematopoietic progenitor cell growth and development (Coppin et al., 2015; Gugasyan et al., 2002). *DOK2* is also associated with colorectal cancer (Wen et al., 2015), lung cancer (Berger et al., 2013; Chen et al., 2019), and renal cancer (Kužma et al., 2019; Solarek et al., 2019). Numerous reports showed that the downregulation of *DOK2* is associated with cancer development (Berger et al., 2010; Coppin et al., 2015; Ohsugi, 2017).

**FIGURE 3:**
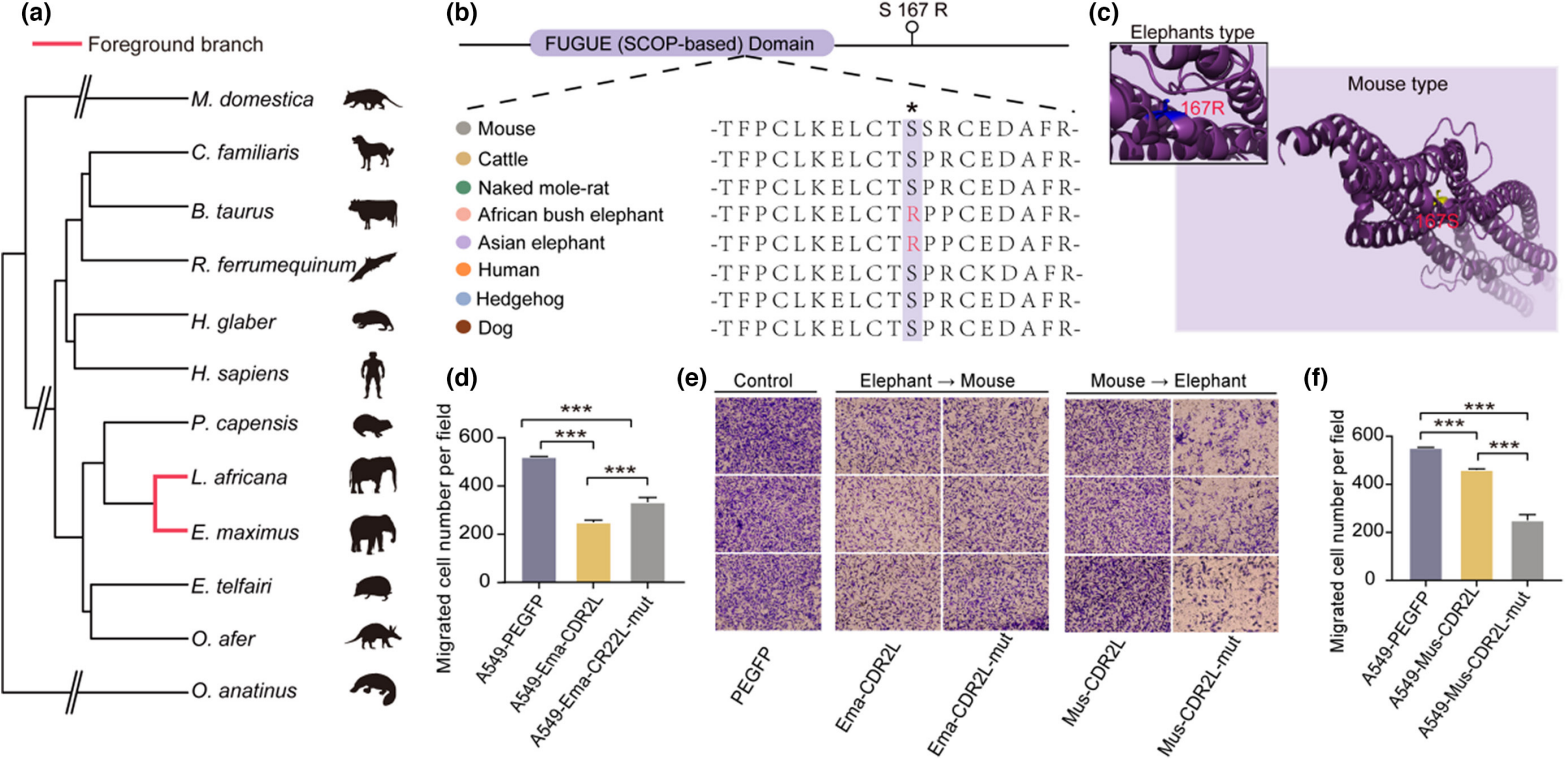
Positively selected genes in Asian elephant and African bush elephant. (a) Phylogenetic tree of 12 mammals in comparative analysis with red line represent the foreground branch (Asian elephant and African bush elephant). Specific mutations of elephants in the *CDR2L* gene. (b) The CDR2L protein sequence of multiple species (indicated with different colors) was aligned. (c) 3D structure simulation of elephants' type compared with that of short‐lived mammals. Functional cellular assays of *CDR2L* gene in short and long‐lived mammals' group. (d) Number of migrated cells of A549‐PEGFP (control), A549‐Ema‐CDR2L (Asian elephant type), and A549‐Ema‐CDR2L‐mut (mouse type) group in the A549 cells. (e) Migration ability of A549‐PEGFP (control) A549‐Ema‐CDR2L (Asian elephant type), A549‐Ema‐CDR2L‐mut (mouse type), A549‐Mus‐CDR2L (mouse type), and A549‐Mus‐CDR2L‐mut (Asian elephant type) group in the A549 cells. (f) Number of migrated cells of A549‐PEGFP (control), A549‐Mus‐CDR2L (mouse type), and A549‐Mus‐CDR2L‐mut (Asian elephant type) group in the A549 cells. *p < 0.05, **p < 0.01, ***p < 0.001, ns: Non‐significant.

One of the interesting PSGs in elephants is *CDR2L* (LRT *p* value = 1.70 × 10^−10^) (Figure 3a), which encodes cerebral degenerated‐related protein 2‐like protein. Actually, both CDR2L and CDR2 are tumor expression antigens, and targets of Yo‐antibodies (Kråkenes et al., 2019). They are associated with a disorder called paraneoplastic cerebellar degradation (PCD), a condition in which the immune system indiscriminately targets tumor antigens, leading to acute cerebellar degradation. CDR2L protein appeared to be the main target of Yo‐antibodies in PCD‐tumors and has been implicated in ovarian and breast cancers (Eichler et al., 2013; Raspotnig et al., 2017, 2022).

We identified one potential positively selected site of *CDR2L* gene in the ancestral lineage leading to Asian elephant and African bush elephant; the residue at position 167 of *CDR2L* gene has a Serine to Argine substitution in two elephants. This substitution is unique to African bush elephant and Asian elephant while the residue of other mammals with whole‐genome resource available is Serine (Figure S7).

We further performed transwell assay to validate the potential ability of residues at positively selected sites (position 167 of the *CDR2L* gene has a Serine to Arginine substitution in two elephants) of *CDR2L* gene (Figure 3b,c). We expressed elephant *CDR2L* gene in A549 cells and measured the in vitro migration ability. The migration assays showed that the number of A549 cells expressing elephant *CDR2L* (Ema‐CDR2L) that migrated through the transwell polycarbonate filter was significantly lower than that of cells expressing the PEGFP control. Then, we replaced the residue at position 167 (Arginine) in elephant *CDR2L* with Serine and found that the number of A549 cells in mutant group (Ema‐CDR2L‐mut) is higher than elephant group (Ema‐CDR2L) but still lower than PEGFP control group (*p* < 0.001) (Figure 3d,e, Figure S8a), suggesting that residue replacement at position 167 of elephant *CDR2L* represents a potential ability to cancer resistance.

In parallel, we generated the plasmid of mouse *CDR2L* (Mus‐CDR2L, position 167 of the *CDR2L* gene is Serine) and plasmid of mouse *CDR2L* mutant (Mus‐CDR2L‐mut, position 167 of the *CDR2L* gene has a replacement from Serine to Arginine) for further assessment. Our results showed that the migration of A549 cells significantly reduced when we expressed the mouse *CDR2L* (Mus‐CDR2L) compared with PEGFP control, and the migrated number of A549 cells is less than those in mouse group (Mus‐CDR2L) when we expressed the mutant mouse group (Mus‐CDR2L‐mut) (*p* < 0.001) (Figure 3e,f, Figure S10a).

Then, we also generated the plasmid of human *CDR2L* (Human‐CDR2L, position 167 of the *CDR2L* gene is Serine) and plasmid of human *CDR2L* mutant (Human‐CDR2L‐mut, position 167 of the *CDR2L* gene has a substitution from Serine to Arginine) for additional validation. Our transwell results showed that the migrated number of A549‐Human‐CDR2L cells was lower than that of PEGFP control cells, although this difference was not statistically significant. Besides, the migrated number of A549 cells in mutant group (Human‐CDR2L‐mut) was significantly lower than that of cells expressing PEGFP control and human *CDR2L* (Human‐CDR2L) (*p* < 0.001) (Figure S11a,b). Altogether, these findings suggest that the substitution of *CDR2L* in elephants can attenuate A549 cells migration.

Furthermore, we performed in vitro tumor sphere formation assay as a surrogate to evaluate the capacity of tumor inhibition in the substitutions of *CDR2L*. We found that the number of tumor sphere formation was significantly reduced when we expressed the elephant *CDR2L* (Ema‐CDR2L) compared with the control group (*p* < 0.05) (Figure S12a,b). Besides, we also expressed the mutant elephant *CDR2L* (position 167 Arginine was replaced with Serine) in A549 cells to evaluate the ability of tumor inhibition. This produced more tumor spheres than the control group (A549) and the elephant *CDR2L* group (Ema‐CDR2L). Similarly, we found that the number of tumor spheres was significantly reduced when expressing mutant mouse (position 167 Serine was replaced with Arginine) compared to expressing mouse CDR2L (Mus‐CDR2L) and control group (A549) (*p* < 0.05) (Figure S12a,b). Altogether, tumor sphere formation assays suggested that the substitution of *CDR2L* in elephants enhanced the tumor inhibition ability.

### 3.4 Positively selected genes in four long‐lived mammals

**FIGURE 4:**
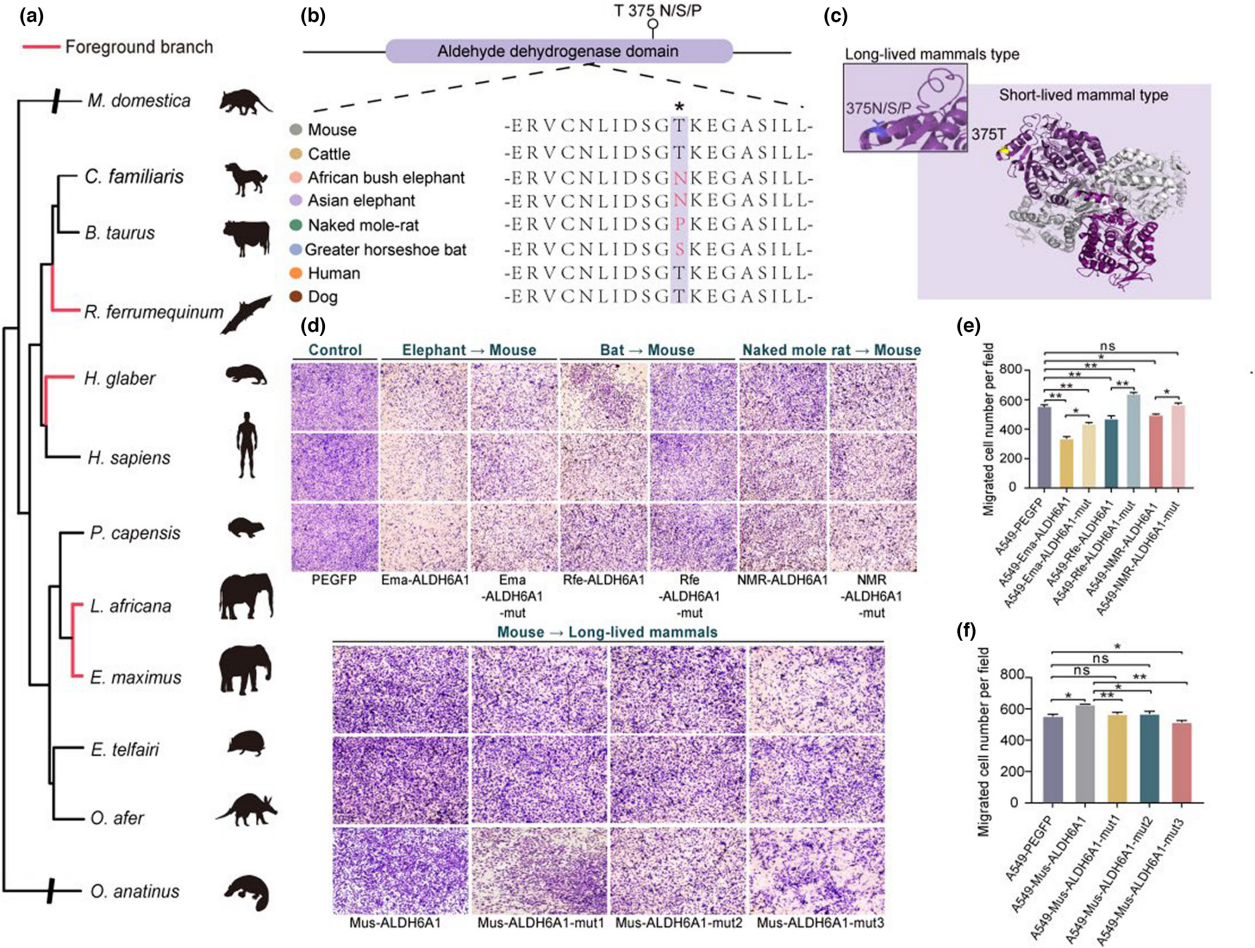
Positively selected genes in long‐lived mammals. (a) Phylogenetic tree of 12 mammals in comparative analysis with red line representing the foreground branch (Asian elephant, African bush elephant, greater horseshoe bat, and naked mole rat). Specific mutations of long‐lived mammals in the *ALDH6A1* gene including one mutation in the Aldehyde dehydrogenase domain. (b) The ALDH6A1 protein sequence of multiple species (indicated with different colors) was aligned. (c) 3D structure simulation of long‐lived mammal's type compared with that of short‐lived mammals. Functional cellular assays of *ALDH6A1* gene in short and long‐lived mammals' group. (d) Migration ability of in the A549‐PEGFP (control), A549‐Ema‐ALDH6A1 (Asian elephant type) and A549‐Ema‐ALDH6A1‐mut (mouse type), A549‐Rfe‐ALDH6A1 (greater horseshoe bat type) and A549‐Rfe‐ALDH6A1‐mut (mouse type), A549‐NMR‐ALDH6A1 (naked mole rat type), A549‐ NMR‐ALDH6A1‐mut (mouse type), A549‐Mus‐ALDH6A1 (mouse type), A549‐Mus‐ALDH6A1‐mut1 (Asian elephant type), A549‐Mus‐ALDH6A1‐mut2 (greater horseshoe bat type) and A549‐Mus‐ALDH6A1‐mut3 (naked mole rat type) group in the A549 cells. (e) Number of migrated cells in the A549‐PEGFP (control), A549‐Ema‐ALDH6A1 (Asian elephant type) and A549‐Ema‐ALDH6A1‐mut (mouse type), A549‐Rfe‐ALDH6A1 (greater horseshoe bat type) and A549‐Rfe‐ALDH6A1‐mut (mouse type), A549‐NMR‐ALDH6A1 (naked mole rat type) and A549‐ NMR‐ALDH6A1‐mut (mouse type) group in the A549 cells. (f) Number of migrated cells migration ability in the A549‐PEGFP (control), A549‐Mus‐ALDH6A1 (mouse type), A549‐Mus‐ALDH6A1‐mut1 (Asian elephant type), A549‐Mus‐ALDH6A1‐mut2 (greater horseshoe bat type) and A549‐Mus‐ALDH6A1‐mut3 (naked mole rat type) group in the A549 cells. *p < 0.05, **p < 0.01, ***p < 0.001, ns: Non‐significant.

To explore genes under positive selection across distant long‐lived taxa, we enlarged our test of foreground branch to include other two long‐lived mammals: naked mole rat and greater horseshoe bat (Figure 4a). The selective pressure analysis found signatures of positive selection on 132 genes in four long‐lived mammals (Table S21). Among them, 79.5% (105 genes) are also under positive selection in the ancestral branch of elephants (Figure S8). These genes are significantly enriched in basal cell carcinoma pathway (*p* value = 0.007), other types of O‐glycan biosynthesis pathway (*p* value = 0.02), gap junction (*p* value = 0.02), and adherens junction (*p* value = 0.03) (Figure S6c,d, Table S22). Basal cell carcinoma (BCC) is one of the most common skin malignancies worldwide (Niculet et al., 2022). The development of BCC is the result of a complex interplays between environmental, phenotypic, and genetic factors, with ultraviolet radiation considered to be the main risk factor (Dika et al., 2020). Previous studies have shown that UV exposure leads to deleterious effects such as skin aging and cancer through generating cellular reactive oxygen species and DNA damage (Yu & Lee, 2017). Several PSGs may involve in tumorigenesis (Table S21). For instance, COP9 signalosome subunit 3 (*COPS3*, LRT *p* value = 1.11 × 10^−6^) is closely associated with tumor development (Both et al., 2016; van Dartel & Hulsebos, 2004) and knockdown of *COPS3* significantly downregulated MEK signaling, reducing metastasis of osteosarcoma cells (Zhang et al., 2018). Guanosine monophosphate reductase (*GMPR*, LRT *p* value = 0.05 × 10^−2^) is another positively selected gene detected in long‐lived mammals, which is a potential tumor suppressor that inhibits the regulatory pathway in tumor cells (Wawrzyniak et al., 2013). We have identified the selective signal in metal responsive transcription factor 1 (*MTF1*, LRT *p* value = 9.85 × 10^−5^), which is upregulated in malignant ovarian cancer and might contribute to ovarian tumor metastasis.

Over recent years, there has been growing interest in genome‐wide sequencing and comparison of long‐lived mammals (Keane et al., 2015; Kim et al., 2011; Seim et al., 2013). Genome sequencing found 45 genes were under positive selection in the naked mole rat (Kim et al., 2011). Interestingly, two of them (*RBM4* and *RBM28*) were detected to be positively selected in four long‐lived mammals by this study. Moreover, signatures of positive selection were also detected from other RNA‐binding motif proteins (RBM), that is, *RBM14* (LRT *p* value = 1.87 × 10^−9^), *RBM27* (LRT *p* value = 0.01 × 10^−2^), and *RBM33* (LRT *p* value = 1.14 × 10^−10^). The RNA‐binding motif proteins are a class of important intracellular proteins, and are associated with the occurrence and development of cancers (Jögi et al., 2009; Zhang et al., 2014). For example, *RBM4* inhibits the apoptosis of breast cancer cells by upregulating the expression of IR‐B and MCL‐1S (Lin et al., 2014) and *RBM33* participates in promoting proliferation in various cancers (Li et al., 2021). Therefore, RBM protein family might serve as the common targets for antagonizing cancer in long‐lived mammals. Vicens and Posada (2018) have assessed the selective pressures on 430 cancer related genes and showed evidence of positive selection on *CSF3R* (Vicens & Posada, 2018), which is the receptor for colony‐stimulating factor 3. Mutations in *CSF3R* are common in patients with chronic neutrophilic leukemia or atypical chronic myeloid leukemia (Maxson et al., 2013), and this gene has been also detected under positive selection in four long‐lived mammals (LRT *p* value = 5.00 × 10^−4^). Seim et al. (2013) have examined the positively selected genes in the longest‐lived bat species, Brandt's bat and found myotilin (*MYOT*) was under positive selection. Our analyses evidenced the signature of selection of this gene (LRT *p* value = 1.67 × 10^−8^) (Table S21). *MYOT* belongs to a small protein family of immunoglobulin (Ig) domain‐containing proteins in the Z‐line associated with the actin cytoskeleton (Otey et al., 2009), where *MYOT* is usually expressed in the heart and involved in muscular dystrophy. Previous observations have shown myotilin to be significantly elevated with age (Han et al., 2022).

### 3.5 Functional assay of common substitutions in *ALDH6A1*

One of the PSGs that attracts our interest is *ALDH6A1* (LRT *p* value = 8.80 × 10^−5^), which encodes mitochondrial methylmalonate semialdehyde dehydrogenase protein (Figure 4b,c). Members of the aldehyde dehydrogenase superfamily (ALDHs) are a group of oxidizing enzymes that function in diverse cellular activities including aldehyde oxidation, detoxification, and antioxidants (Jackson et al., 2011; Marchitti et al., 2008; Shortall et al., 2021; Vasiliou & Nebert, 2005). Deficiencies or mutations in ALDH have been implicated in various forms of cancer and metabolic disorders (Lu et al., 2020; Xu et al., 2015; Yu et al., 2010). *ALDH6A1* is reported to be regulated in several processes of cancer, including hepatocellular carcinoma and prostate cancer, and diabetes (Cho et al., 2018; Dharuri et al., 2014; Lu et al., 2020). Specifically, *ALDH6A1* is regulated by a well‐known suppressor transcription factor HNF4A, which suppressed tumorigenic capability in clear renal cell carcinoma (Lu et al., 2020). Another study showed that inhibition of *ALDH6A1* may be strongly associated with abnormal proliferation of liver cancer cells (Shin et al., 2020).

We detected one positively selected site of *ALDH6A1* in four long‐lived mammals, and we found that the residue of the positively selected site is different in long‐lived mammals. The residue of elephants, greater horseshoe bat, and naked mole rat are respectively Asparagine (N), Serine (S), and Proline (P) while the residue of most mammals is Threonine (T) (Figure S9). This residue is specific in elephants compared to other mammals, while the residue of greater horseshoe bat is also unique when compared to other bats with the Threonine (T) residue. We also found guinea pig also have Asparagine at the site, which is identical to naked mole rate and might be related to the close phylogenetic relationship between these two species.

We then generated plasmids that express *ALDH6A1* of four long‐lived mammals (Asian elephant and African bush elephant: Ema‐ALDH6A1, greater horseshoe bat: Rfe‐ALDH6A1, naked mole rat: NMR‐ALDH6A1) and mouse (Mus‐ALDH6A1). As we expected, the A549 cells that with overexpressed long‐lived mammals *ALDH6A1* showed inhibited migration compared to A549 cells transfected with the PEGFP control and mouse *ALDH6A1* (Figure 4d,e, Figure S10b). This not only evidenced that *ALDH6A1* overexpression could inhibit growth and migration of cancer cells (Cai et al., 2022), but also indicated the *ALDH6A1* of long‐lived mammals show stronger inhibition of cellular migration of cancer cells. However, there are some subtle differences in migration ability among cells expressed four long‐lived mammals *ALDH6A1*. In particular, the migration of A549 cells that expressed elephants *ALDH6A1* is less than A549 cells expressed greater horseshoe bat and naked mole rat *ALDH6A1*, suggesting the elephant *ALDH6A1* has the highest inhibition effects. Next, we created three mutants in *ALDH6A1* of long‐lived mammals for additional validation, that is, Ema‐ALDH6A1‐mut, Rfe‐ALDH6A1‐mut, and NMR‐ALDH6A1‐mut, by replacing residues under positive selection in long‐lived mammals into mouse residue. Expectedly, we observed a higher cell migration in A549 cells transfected with these mutants, providing additional validation on the role of positively selected sites in long‐lived mammals. In vice versa, three plasmids' (Mus‐ALDH6A1‐mut1, Mus‐ALDH6A1‐mut2, Mus‐ALDH6A1‐mut3) mutants were also generated using mouse *ALDH6A1* plasmid by substituting residue at the site of selection to residue of the long‐lived mammals. Interestingly, A549 cells transfected with these mutants have shown suppressed cell migration than the Mus‐ALDH6A1 group (Figure 4d,f, Figure S10b).

Besides, we also generated plasmids that express *ALDH6A1* of human (A549‐Human‐ALDH6A1) for the transwell assays. As we expected, the A549 cells with overexpressing human *ALDH6A1* showed inhibited migration compared to A549 cells transfected with the PEGFP control (A549‐PEGFP). Furthermore, three plasmids' (Human‐ALDH6A1‐mut1, Human‐ALDH6A1‐mut2, Human‐ALDH6A1‐mut3) mutants were also generated using human *ALDH6A1* plasmid by substituting residue at the site of selection to residue of the long‐lived mammals. The transwell results showed that A549 cells transfected with these mutants have shown suppressed cell migration than the Human‐ALDH6A1 group. However, there are also some subtle differences in migration ability among cells expressed three mutants. In particular, the migration of A549 cells that expressed Human‐ALDH6A1‐mut1 (Asian elephant type) is less than A549 cells that expressed Human‐ALDH6A1‐mut2 (greater horseshoe bat type) and Human‐ALDH6A1‐mut3 (naked mole rat type) (Figure S11a,c), suggesting the elephant *ALDH6A1* have the higher inhibition effects which is consisted with the transwell results of A549 cells overexpressing long‐lived mammals *ALDH6A1*.

Taken together, our results reveal that the common residues at positively selected sites in *ALDH6A1* of long‐lived mammals offer an enhanced function in resisting cancer progression.

## 4 DISCUSSION

One of the major restrictions on the evolution of large body sizes across mammals is the high risk of cancer incidence due to the hypothesis that the increase in cell division may bring an increasement cancer risk. However, there is no correlation between large body size and the risk of cancer development. Therefore, large mammals might have evolved enhanced cancer resistance mechanisms to suppress the cancer incidence and extend their lifespans as well (Omotoso et al., 2021). For example, elephants are the mammals with large body size (~5500 kg); however, recent studies show that elephants are resistant to cancer, with an estimated cancer mortality rate of 4.81%, compared to 11%–25% for humans (Abegglen et al., 2015). At the same time, multiple copies of TP53, most of them are pseudocopies, were found in elephant genome (Abegglen et al., 2015; Sulak et al., 2016). Subsequent study further found refunctionalizing of another pseudogene, LIF, may mediate the cancer resistance and body size in elephants (Vazquez et al., 2018). Given the long generation time of elephants, these results implied that the expanded pseudocopies of cell cycle check‐point genes are key responses to cancer resistance in elephants. In this study, by performing gene family analysis in the ancestral lineage of elephants, we found genes involved in several other pathways, such as Ras‐associated protein‐1 (Rap1) signaling pathway and base excision repair pathway, were also expanded in elephant genomes. This could relate to cancer resistance because base excision repair pathway is the predominant repair pathway in mammalian cells and is a critical process of genome maintenance, with cancer, premature aging and metabolic disorders, were observed in animals lacking base excision repair function (Mostoslavsky et al., 2006; Vartanian et al., 2006). In addition, several positively selected genes, for example, *CDR2L*, might also involve cancer resistance in elephants. These analyses and results would indicate the large body size and low tumor incidence in elephant is more complex than solely on contributions from pseudogenes.

Another interesting question is whether such cancer resistance in diverse long‐lived lineages could be achieved by common approach, that is, “convergent” evolution. In this study, we screened positive selection genes and examined the cellular effect of common substitutions in *ALDH6A1* of long‐lived mammals, hence provide a piece of evidence that cancer‐resistant substitutions can be utilized by long‐lived mammals. However, this does not mean that tumor resistance is convergently evolved in long‐lived mammals as it is unknown what are the common benefits brought by such “convergent” phenotype. Moreover, the results could be challenged by including more long‐lived or short‐lived species. This is also understandable as there is no consistent definition for “long‐lived,” or strong correlation between longevity and tumor incidence. Nevertheless, the analyses and results presented here could serve as a preliminary survey of common substitutions response to cancer resistance in long‐lived animals.

## AUTHOR CONTRIBUTIONS

X.Z. conceived the study and designed the project. X.L. and P.W. performed experiments, completed analysis, and wrote the manuscript; J.D., Z.L., and M.L., prepared the cell cultures; Q.P. implement the data analysis and generated structural figures; X.L., P.W., Q.P., G.L., O.O., W.L., and X.Z. discussed the results and revised this manuscript; all authors contributed to data interpretation.

## CONFLICT OF INTEREST STATEMENT

The authors declare no competing financial interests.

## Supporting information


Appendix S1.

Table S19.

Table S21.

## Data Availability

Genome and RNA sequencing data from this study have been deposited in BioProject at NCBI (PRJCA013758), and the Genome Sequence Archive in National Genomics Data Center, China, National Center for Bioinformation/Beijing Institute of Genomics, Chinese Academy of Sciences (CRA009178).
