# Supplementary material for: Chromosome‐level Asian elephant genome assembly and comparative genomics of long‐lived mammals reveal the common substitutions for cancer resistance
Source: Aging Cell. 2023 Jul 3;22(9):e13917. doi: 10.1111/acel.13917 (PMC10497851; doi:10.1111/acel.13917)
Supplement: Supplementary file 1 — Appendix S1. [file ACEL-22-e13917-s002.docx]

Supplementary Information for

**Chromosome-level Asian elephant genome assembly and comparative genomics of long-lived mammals reveal the common substitutions for cancer resistance**

Xuanjing Li, Pengcheng Wang, Qi Pan, Gaoming Liu, Weiqiang Liu, Olatunde Omotoso, Juan Du, Zihao Li, Yang Yu, Yun Huang, Pingfen Zhu, Meng Li, Xuming Zhou

Correspondence to: zhouxuming@ioz.ac.cn

**This PDF file includes:**

Figs. S1 to S8

Tables S1 to S22

**Other Supplementary Materials for this manuscript include the following (separate files):**

Tables S19

Tables S21

**Supplementary Figures**


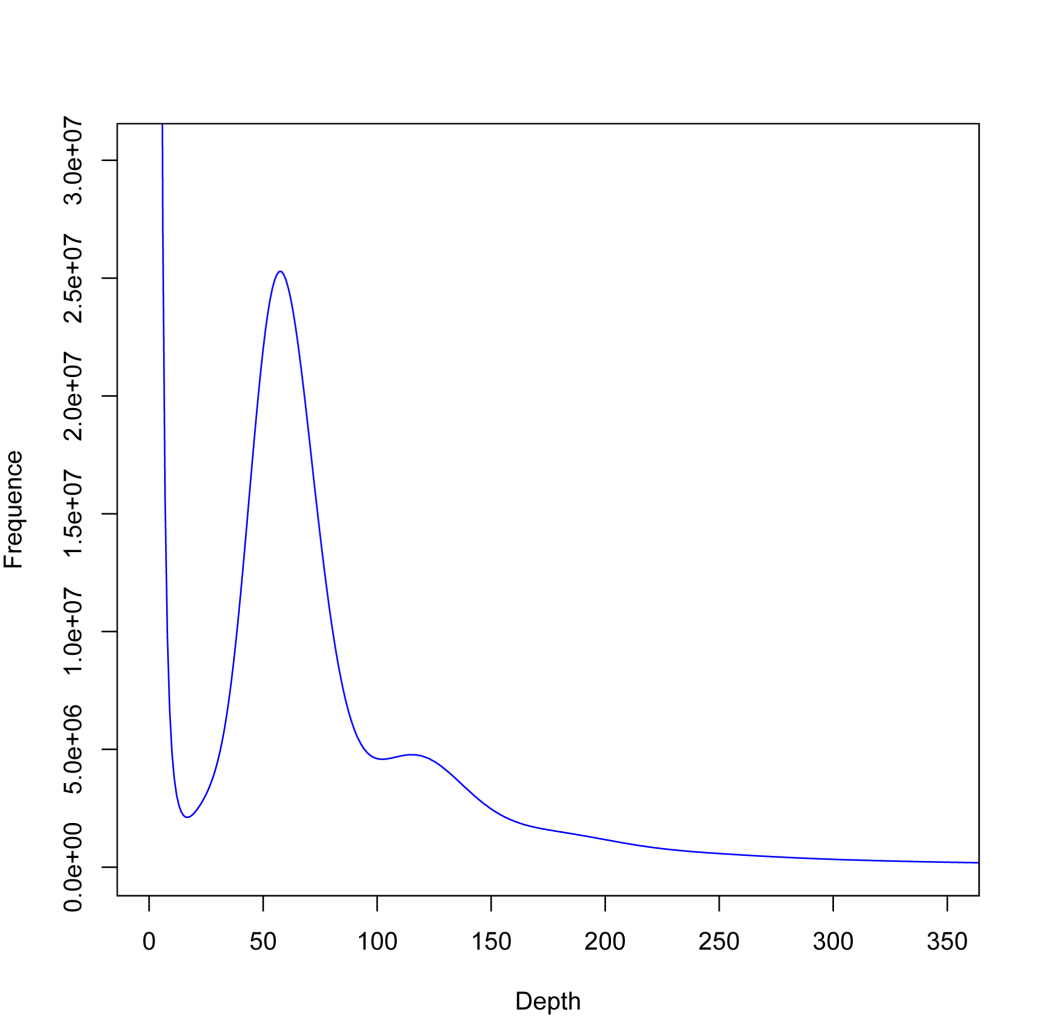


**Supplementary Figure 1. The distribution of 17-mer in Asian elephant genome.** The X-axis is the depth of K-mers derived from the sequenced reads and the Y-axis is the frequency of the K-mer depth.

**.**


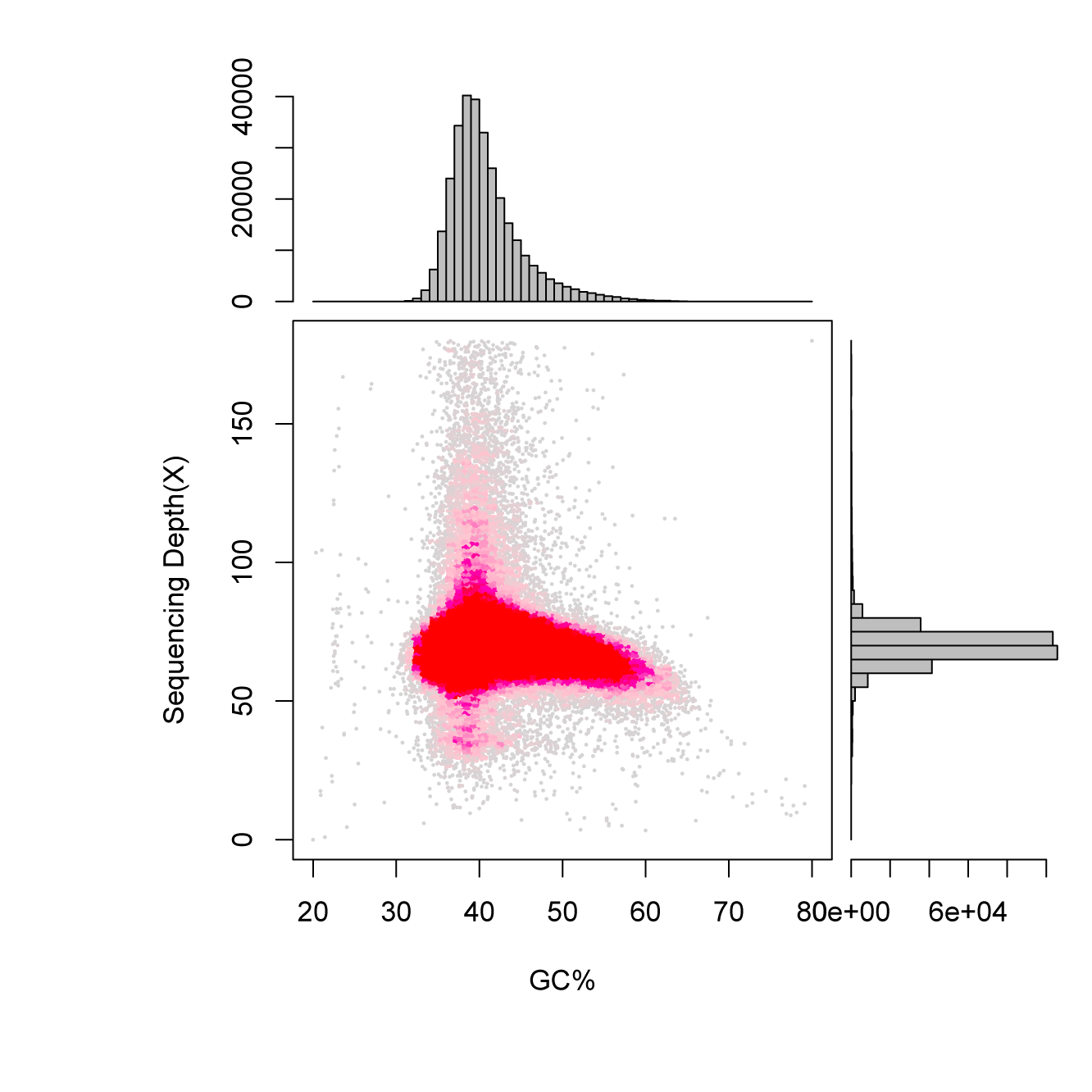


**Supplementary Figure 2. The relationship between GC content and sequencing depths in Asian elephant assembly.** The x-axis represents GC content (%), and y-axis represents the average depth. The 10 kb non-overlapping sliding windows was used when the GC content and average depth were calculated.


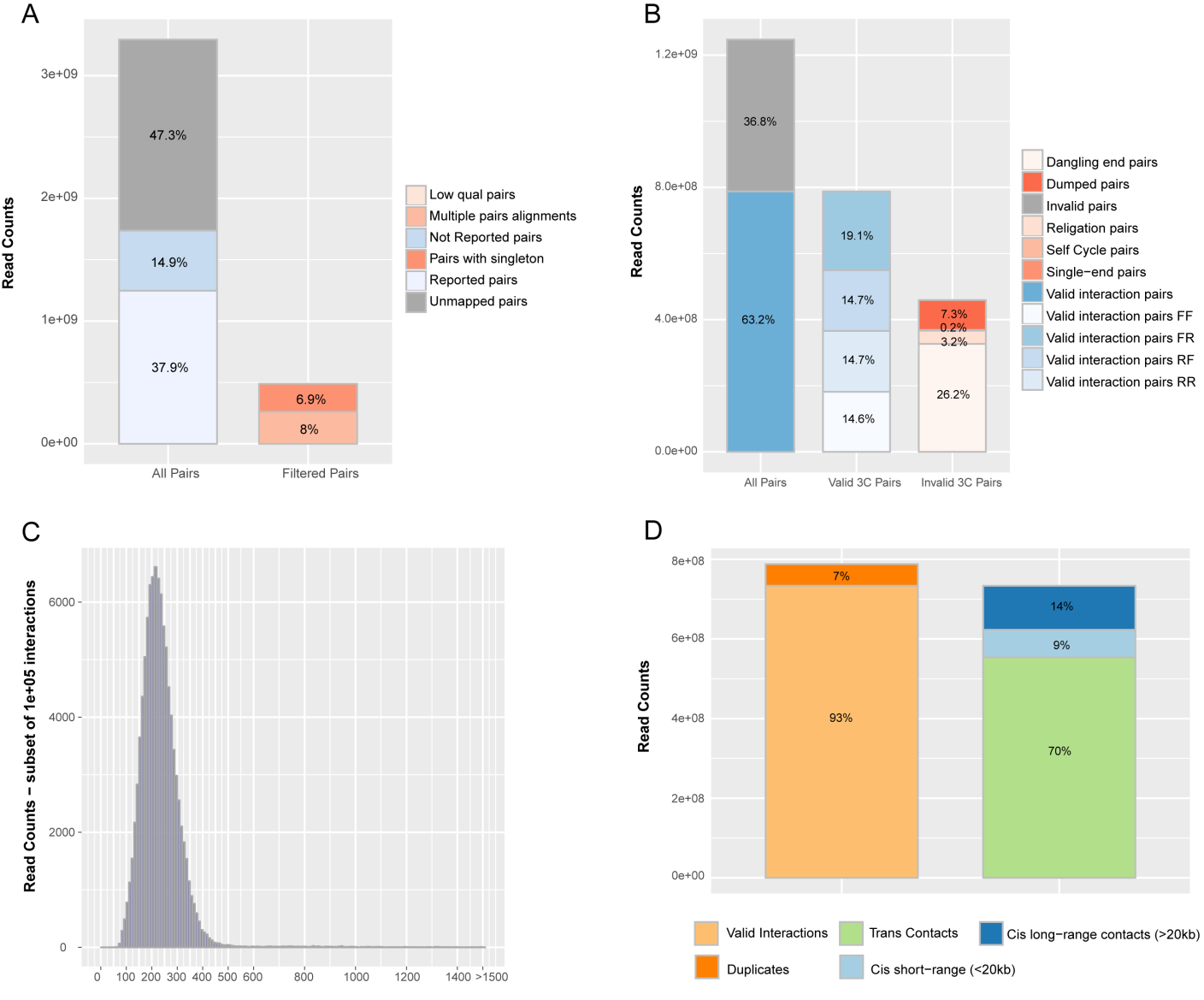


**Supplementary Figure 3. Summary statistics of Hi-C analysis for Asian elephant genome. (A)** Statistics after read pairing; **(B)** Statistics of read pairs alignment on restriction fragments; **(C)** Valid pairs − fragment size distribution; **(D)** Valid pairs − duplicates and contact ranges.


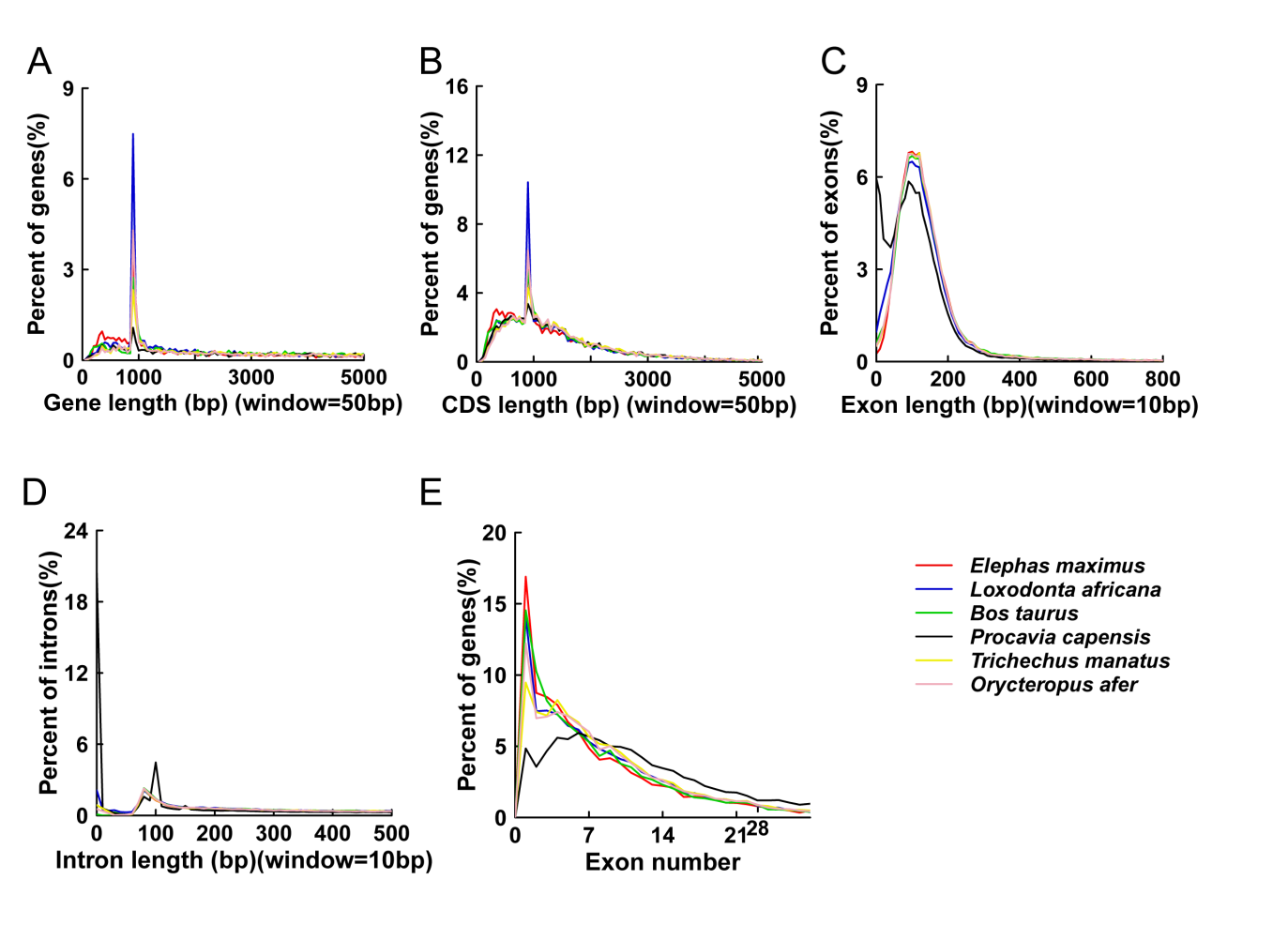
**Supplementary Figure 4. Evaluation of gene annotations.** Distribution of mRNA length (**A**), distribution of CDS length (**B**), distribution of exon length (**C**), distribution of intron length (**D**), and distribution of exon number (**E**) per gene among coding genes of *E. maximus*, *L. Africana*, *B. Taurus*, *P. capensis*, *T. manatus* and *O.afer* genomes are presented. The x-axes indicate length or numbers of genes, mRNA, CDS, exons or introns, and y-axes represent the percent of genes, exons or introns. The similarities between these variables of our Asian elephant genome and other mammals’ genomes indicate that our assembly and annotation possess high quality.


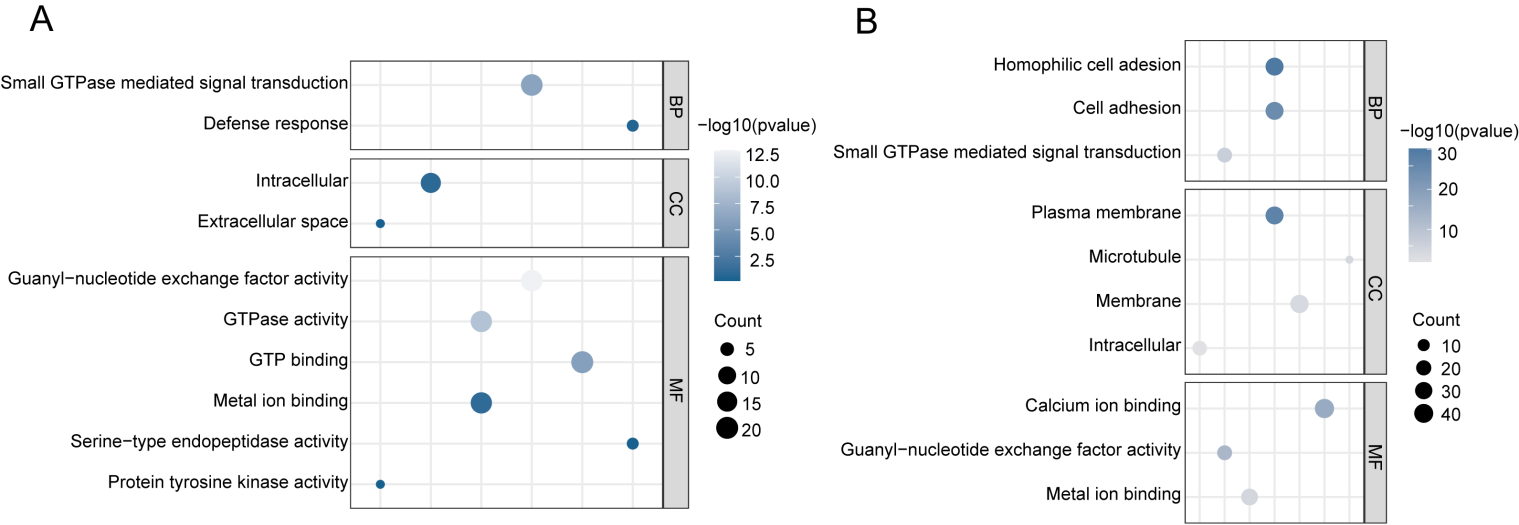


**Supplementary Figure 5. Functional enrichment analysis of expanded gene families in Asian elephant and African bush elephant. (A)** GO analysis for the expanded gene families in the ancestral branch of Asian elephant and African bush elephant; **(B)** GO analysis for the expanded gene families in Asian elephant. Dot color indicates *p*-values and dot size indicates the count.

**Supplementary Figure 6. Functional enrichment analysis of positively selected genes (PSGs) in Asian elephant, African bush elephant, naked-mole rat and greater horseshoe bat (A-D). (A)** GO and **(B)** KEGG analysis for PSGs identified in Asian elephant and African bush elephant; **(C)** GO and **(D)** KEGG analysis for PSGs identified in four long-lived mammals: Asian elephant, African bush elephant, naked-mole rat and greater horseshoe bat.


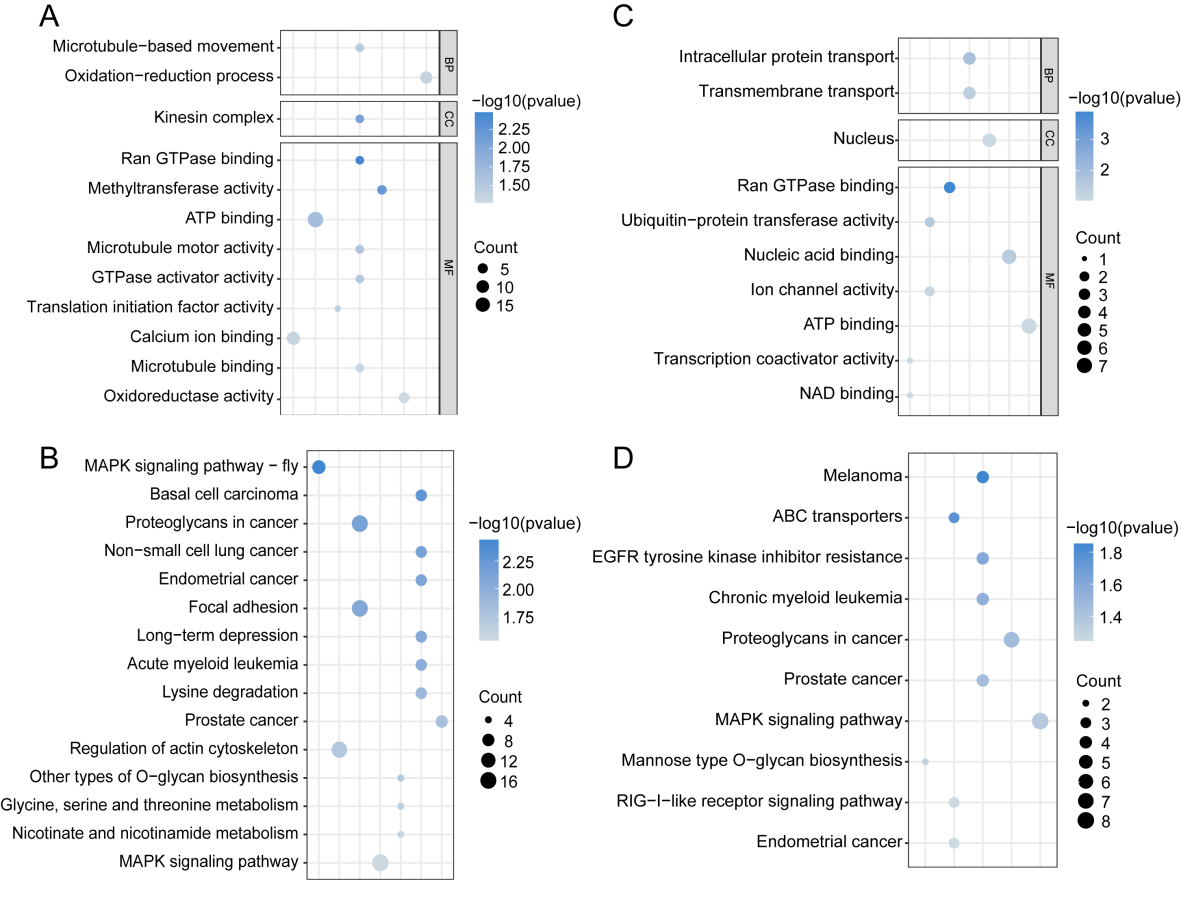

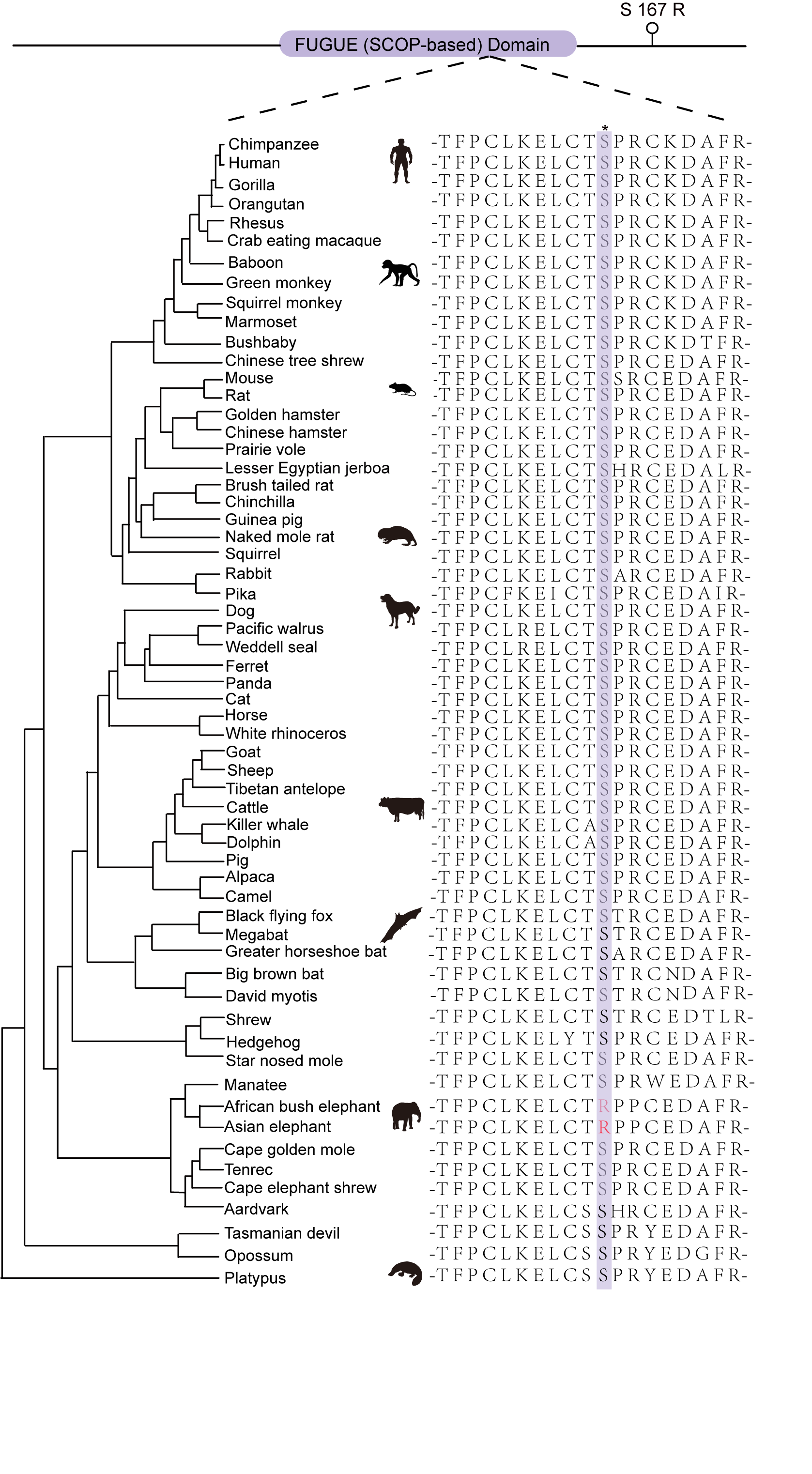


**Supplementary Figure 7. The alignment of *CDR2L* gene in all mammals with genome available.**


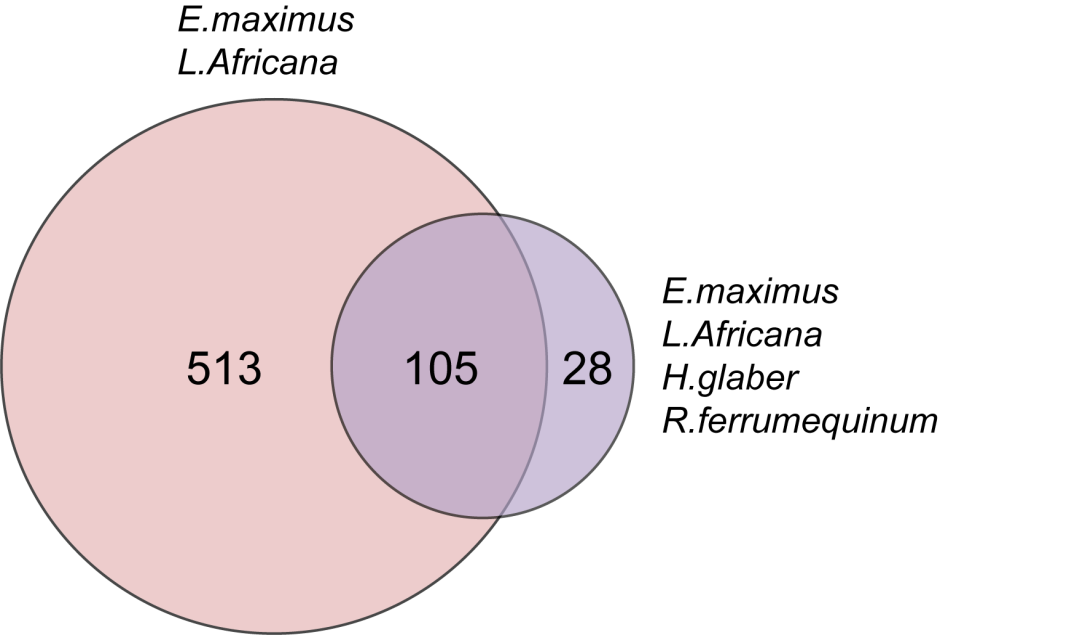


**Supplementary Figure 8.** A Venn diagram showing the number of genes found to be under positive selection in the two foreground branch settings (*E.maximus+L.Africana, E. maximus* +*L. Africana* +*H. glaber*+*R. ferrumequinum*) tests and how many overlapped between two tests.

**
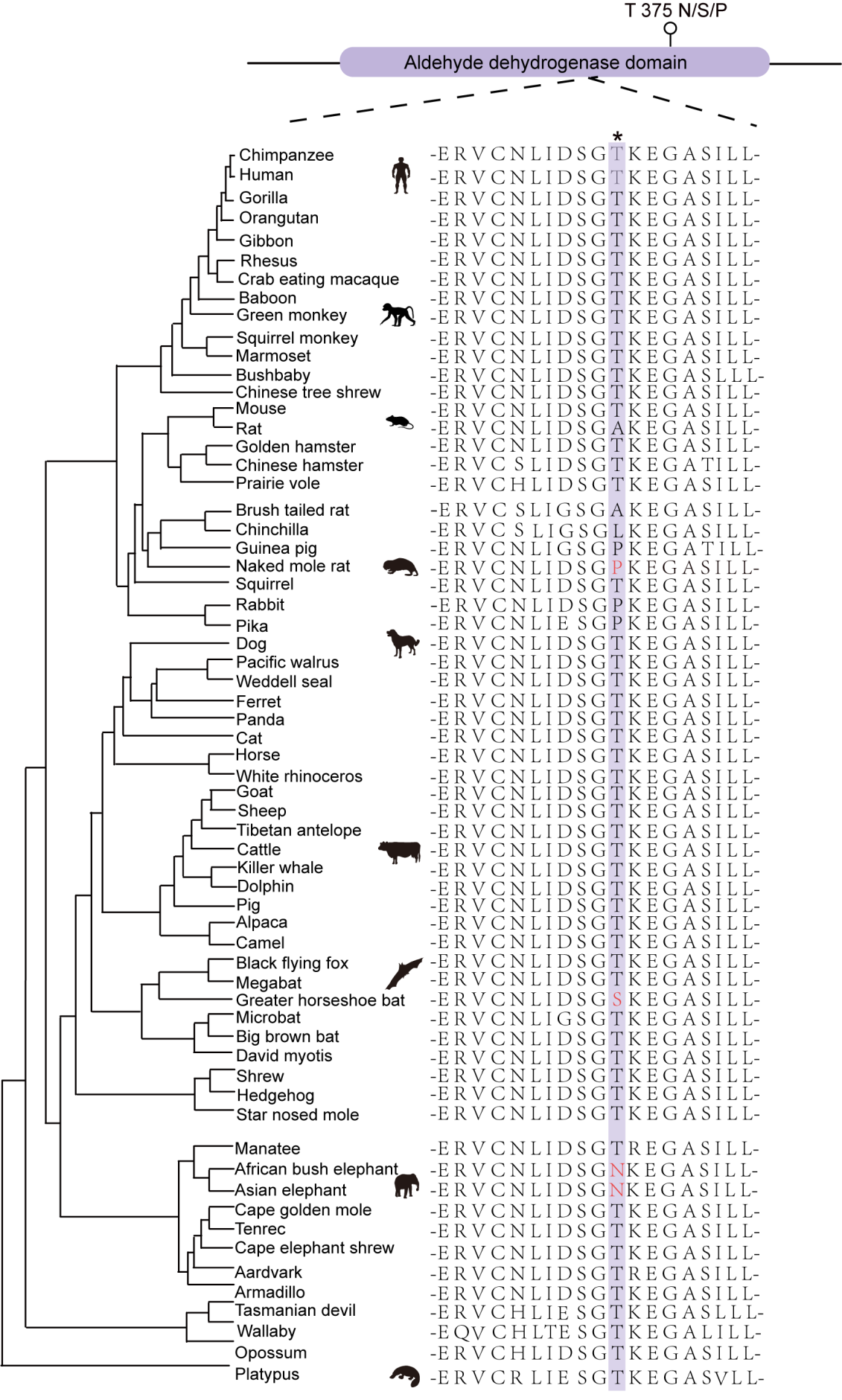
**

**Supplementary Figure 9. The alignment of *ALDH6A1* gene in all mammals with genome available.**


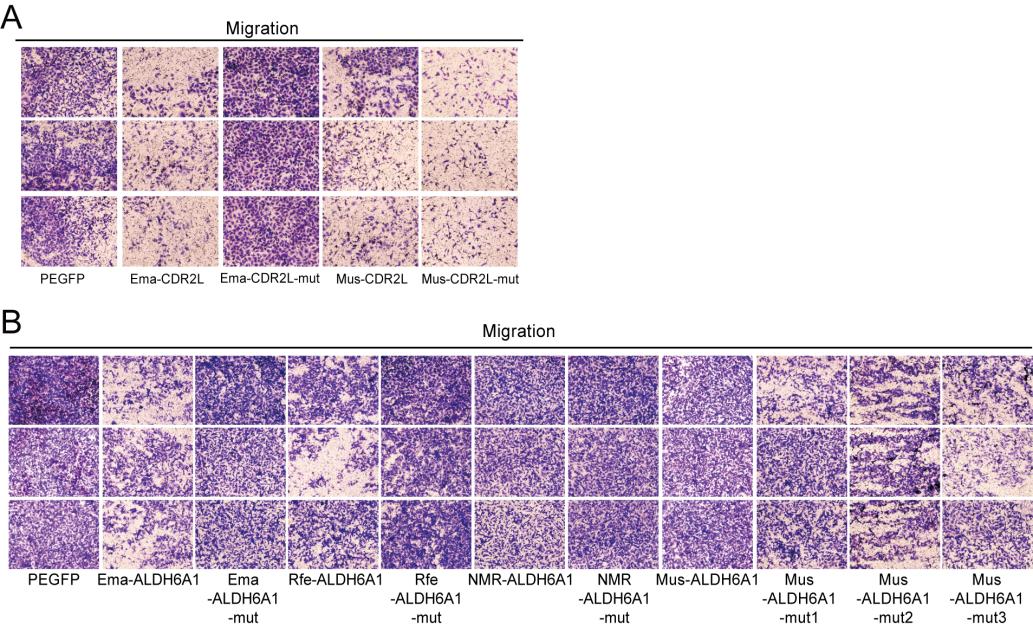


**Supplementary Figure 10. Functional cellular assays of *ALDH6A1* and *CDR2L* gene in short and long-lived mammals’ group.** Effects of *ALDH6A1* gene on A549 cells migration. **(A)** Migration ability of PEGFP (control), Ema-CDR2L (Asian elephant type) and Ema-CDR2L-mut (mouse type), Mus-CDR2L (mouse type) and Mus-CDR2L-mut (Asian elephant type) group in the A549 cells. **(B)** Migration ability of in the PEGFP (control), Ema-ALDH6A1 (Asian elephant type) and Ema-ALDH6A1-mut (mouse type), Rfe-ALDH6A1 (greater horseshoe bat type) and Rfe-ALDH6A1-mut (mouse type), NMR-ALDH6A1 (naked-mole rat type) and NMR-ALDH6A1-mut (mouse type), Mus-ALDH6A1 (mouse type), Mus-ALDH6A1-mut1(Asian elephant type), Mus-ALDH6A1-mut2 (greater horseshoe bat type) and Mus-ALDH6A1-mut3 (naked-mole rat type) group in the A549 cells.


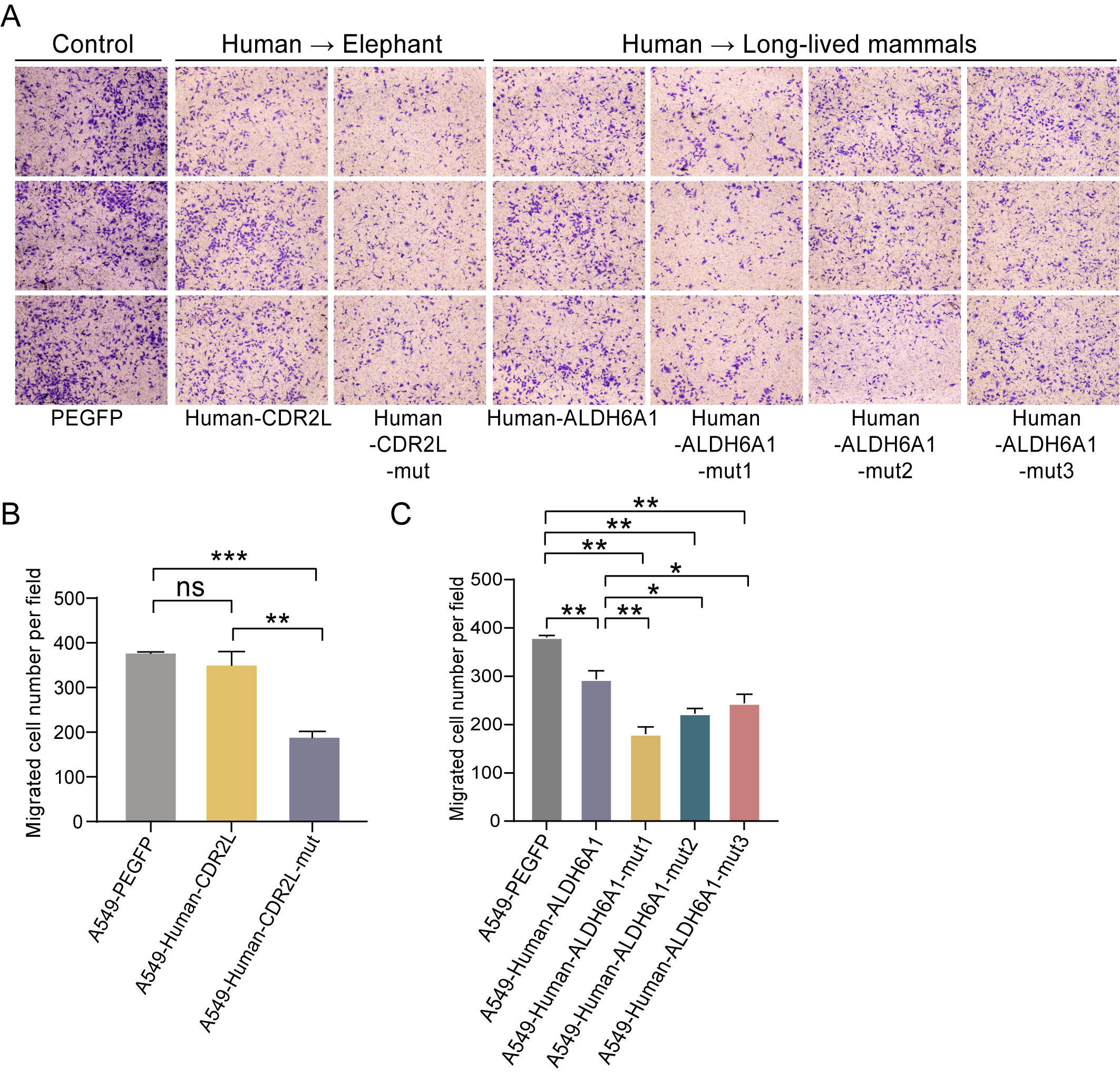


**Supplementary Figure 11. Functional cellular assays of *CDR2L* and *ALDH6A1* gene in human and human mutant group. (A)** Migration ability of PEGFP (control group), Human-CDR2L (human type) and Human-CDR2L-mut (Asian elephant type), Human-ALDH6A1 (human type), Human-ALDH6A1-mut1 (Asian elephant type), Human-ALDH6A1-mut2 (greater horseshoe bat type) and Human-ALDH6A1-mut3 (naked mole rat type) in the A549 cells. **(B)** Number of migrated cells of PEGFP (control group), Human-CDR2L (human type) and Human-CDR2L-mut (Asian elephant type) in the A549 cells. **(C)** Number of migrated cells of PEGFP (control group), Human-ALDH6A1 (human type), Human-ALDH6A1-mut1 (Asian elephant type), Human-ALDH6A1-mut2 (greater horseshoe bat type) and Human-ALDH6A1-mut3 (naked mole rat type) in the A549 cells. **P*<0.05, ** *P*<0.01, *** *P*<0.001, ns: Non-significant.

**
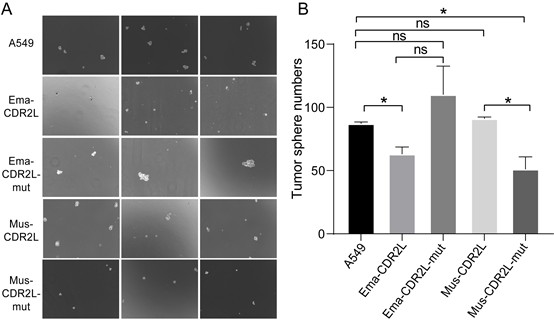
Supplementary Figure 12. The substitution of *CDR2L* inhibits tumor sphere formation in A549 cells. (A)** Representative field of tumor spheres in A549 cells. **(B)** The number of tumor spheres formed by expressing Ema-CDR2L, Ema-CDR2L-mut, Mus-CDR2L and Mus-CDR2L-mut. **P*<0.05, ***P*<0.01, ****P*<0.001, ns: Non-significant. Red arrow indicates the representative tumor sphere.

**Supplementary Tables**

**Supplementary Table S1.** The estimation of Asian elephant genome size using 17-mer analysis.

| **K-mer** | **K-mer number** | **Pkdepth** | **Genome size (M)** | **Used_base** | **Depth** |
| --- | --- | --- | --- | --- | --- |
| 17 | 212,117,653,272 | 60 | 3,535.29 | 253 Gb | 71 |

Notes, Genome Size (Mbp): Genome size = k-mer number/K-mer Depth

**Supplementary Table S2.** Mapping ratio of reads of Asian elephant genome statistics.

| **Statistics** | **Results** |
| --- | --- |
| Reading mapping rate (%) | 99.48 |
| Genome average sequencing depth (×) | 77.88 |
| Coverage of genome (%) | 99.99 |
| Coverage of genome > 4× (%) | 99.95 |
| Coverage of genome > 10× (%) | 99.85 |
| Coverage of genome > 20× (%) | 99.56 |

Notes: The result indicates that our assembly had high quality.

**Supplementary Table S3.** Statistics of the reads in Asian elephant genome using Hi-C analysis

| **Raw reads pairs** | **Valid interactions** | **Valid pairs percentage** | **Valid reads pairs** |
| --- | --- | --- | --- |
| 3,294,037,451 | 93% | 22.27% | 733,577,314 |

**Supplementary Table S4.** Summary statistics of assembled chromosome-level giraffe genome

| **Chromosome ID** | **Length (bp)** | **Percentage (%)** |
| --- | --- | --- |
| chr1 | 237,448,774 | 7.36 |
| chr2 | 231,364,587 | 7.17 |
| chr3 | 201,029,069 | 6.23 |
| chr4 | 188,383,811 | 5.84 |
| chr6 | 162,256,676 | 5.03 |
| chr7 | 141,021,845 | 4.37 |
| chr8 | 132,122,935 | 4.09 |
| chr9 | 123,353,805 | 3.82 |
| chr10 | 118,153,099 | 3.66 |
| chr11 | 111,625,164 | 3.46 |
| chr12 | 108,259,516 | 3.35 |
| chr13 | 103,464,592 | 3.21 |
| chr14 | 93,044,542 | 2.88 |
| chr15 | 92,307,362 | 2.86 |
| chr16 | 84,042,526 | 2.60 |
| chr17 | 82,312,594 | 2.55 |
| chr18 | 82,193,621 | 2.55 |
| chr19 | 79,975,359 | 2.48 |
| chr20 | 78,663,377 | 2.44 |
| chr21 | 78,474,792 | 2.43 |
| chr22 | 76,317,945 | 2.36 |
| chr23 | 75,230,220 | 2.33 |
| chr24 | 73,467,117 | 2.28 |
| chr25 | 63,964,875 | 1.98 |
| chr26 | 63,794,634 | 1.98 |
| chr27 | 50,033,143 | 1.55 |
| chr28 | 29,558,466 | 0.92 |
| Total | 3,134,653,320 | 97.12 |
| Unkown | 92,860,869 | 2.88 |

**Supplementary Table S5.** Quality evaluation of the assembled genome using BUSCO software with “mammalia_odb9 database” dataset.

| **Statistics** | **Gene numbers** | **Percentage (%)** |
| --- | --- | --- |
| Complete BUSCOs | 3,862 | 94.1 |
| Complete Single-Copy BUSCOs | 3,847 | 93.7 |
| Complete Duplicated BUSCOs | 15 | 0.4 |
| Fragmented BUSCOs | 136 | 3.3 |
| Missing BUSCOs | 106 | 2.6 |
| Total BUSCO groups searched | 4,104 | 100 |

**Supplementary Table S6.** Repeat elements annotation of the Asian elephant genome

| **Type** | **Repeat Size (bp)** | **Percent of genome (%)** |
| --- | --- | --- |
| Trf | 29,898,856 | 0.93 |
| Repeatmasker | 1,344,099,843 | 41.65 |
| Proteinmask | 537,054,480 | 16.64 |
| De novo | 1,549,618,065 | 48.01 |
| Total | 1,671,979,332 | 51.80 |

Notes: Trf, Tandem Repeat Finder.

**Supplementary Table S7.** Statistics of transposable elements in Asian elephant genome.

|  | **Repbase TEs** | | **Protein TEs** | | **Denovo TEs** | | **Denovo TEs** | |
| --- | --- | --- | --- | --- | --- | --- | --- | --- |
| **Type** | Length(bp) | Percentage of genome (%) | Length(bp) | Percentage of genome (%) | Length(bp) | Percentage of genome (%) | Length(bp) | Percentage of genome (%) |
| DNA | 41,383,812 | 1.28 | 1,054,815 | 0.03 | 16.64 | 0.10 | 42,033,151 | 1.30 |
| LINE | 992,608,997 | 30.76 | 527,755,483 | 16.35 | 1,335,864,871 | 41.39 | 1,535,842,422 | 47.59 |
| SINE | 143,698,086 | 4.45 | 0 | 0.00 | 4,235,675 | 0.13 | 146,368,984 | 4.54 |
| LTR | 170,826,525 | 5.29 | 8,399,662 | 0.26 | 233,744,341 | 7.24 | 367,429,121 | 11.38 |
| Other | 191 | 0.00 | 0 | 0.00 | 0 | 0.00 | 191 | 0.00 |
| Unknown | 0 | 0.00 | 0 | 0.00 | 102,476,775 | 3.18 | 102,476,775 | 3.18 |
| Total | 1,344,099,843 | 41.65 | 537,054,480 | 16.64 | 1,547,043,534 | 47.93 | 1,656,312,609 | 3.18 |

Notes: TE means transposable element; LINE means long interspersed nuclear elements; SINE means short interspersed nuclear elements; LTR means long terminal repeated; Others indicates that the repetitive sequence can be classified by RepeatMasker, but does not belong to the above categories; unknown indicates that the repetitive sequence cannot be classified by RepeatMasker.

**Supplementary Table S8.** Statistics of non-coding RNA.

| **Type** | **Number** | **Length(bp)** | **Percentage of genome (%)** |
| --- | --- | --- | --- |
| miRNA | 2,429 | 190,056 | 0.006 |
| rRNA | 312 | 40,593 | 0.001 |
| snRNA | 1,284 | 142,258 | 0.004 |
| tRNA | 21,499 | 1,541,031 | 0.048 |

Notes: miRNA: micro RNA, rRNA: ribosomal RNA, snRNA: small nuclearRNA, tRNA: transfer RNA

**Supplementary Table S9.** Statistics of annotated genes from the homolog, *de novo*, and transcript.

| **Methods** | **Gene set** | **Gene number** | **Gene length**  **(bp)** | **CDS number** | **Intron length**  **(bp)** | **Exon length (bp)** | **Exon per gene** |
| --- | --- | --- | --- | --- | --- | --- | --- |
| Homolog | *B. taurus* | 23,244 | 52,052.32 | 1,456.52 | 1,456.52 | 189.21 | 189.21 |
|  | *L. africana* | 23,071 | 32,693.46 | 1,437.08 | 4,629.96 | 185.41 | 7.75 |
|  | *O.afer* | 21,571 | 45,669.65 | 1,556.32 | 5,994.28 | 186.18 | 8.36 |
|  | *P. capensis* | 17,199 | 47,822.71 | 1,587.77 | 1,587.77 | 186.01 | 8.54 |
|  | *T. manatus* | 21,297 | 48,463.94 | 1,602.57 | 6,049.22 | 183.22 | 8.75 |
|  | *H.sapiens* | 20,440 | 54,320.94 | 1,625.78 | 6,680.28 | 182.92 | 8.89 |
|  | *M. musculus* | 20,520 | 53,105.67 | 1,556.85 | 7,018.94 | 186.58 | 8.34 |
| Denovo | Augustus | 22,248 | 57,291.62 | 1,497.41 | 7,199.55 | 171.14 | 8.75 |
|  | Genscan | 51,335 | 42,249.43 | 1,155.05 | 6,797.16 | 163.93 | 7.0 |
| Transcript | Hisat+Stringtie | 48,215 | 18,982.90 | 932.48 | 4,428.54 | 183.71 | 5.08 |
| Glean |  | 21,246 | 41,669.69 | 1,593.09 | 5,205.71 | 183.14 | 8.70 |

**Supplementary Table S10.** Functional annotation of Asian elephant genes

| **Database** | **Number** | **Percentage (%)** |
| --- | --- | --- |
| Total | 21,955 | 100 |
| Swissprot | 20,433 | 93.07 |
| KEGG | 18,596 | 84.70 |
| TrEMBL | 20,722 | 94.38 |
| Interpro | 20,308 | 92.50 |
| Overall | 20,767 | 94.59 |

**Supplementary Table S11.** Statistical analysis of gene family clustering results.

| **Species** | **Genes number** | **Unclustered genes** | **Family number** | **Unique families** | **Average genes per family** |
| --- | --- | --- | --- | --- | --- |
| *B. taurus* | 21,539 | 1,590 | 15,940 | 112 | 1.25 |
| *C. familiaris* | 19,992 | 1,561 | 15,193 | 43 | 1.21 |
| *E. maximus* | 21,955 | 3,164 | 15,792 | 81 | 1.19 |
| *E. telfairi* | 16,560 | 2,006 | 12,533 | 76 | 1.16 |
| *H. glaber* | 20,728 | 2,862 | 14,414 | 94 | 1.24 |
| *H. sapiens* | 19,150 | 407 | 15,622 | 71 | 1.20 |
| *L. africiana* | 20,015 | 1,103 | 15,743 | 65 | 1.20 |
| *M. domestica* | 21,371 | 1,992 | 14,413 | 163 | 1.34 |
| *O. afer* | 18,744 | 820 | 15,337 | 44 | 1.17 |
| *O. anatinus* | 21,676 | 6,105 | 12,024 | 136 | 1.29 |
| *P.capensis* | 15,994 | 984 | 13,246 | 16 | 1.13 |
| *R. ferrumequinum* | 19,529 | 1,359 | 15,488 | 37 | 1.17 |

**Supplementary Table S12.** Expanded and contracted gene families on branches.

| **Species** | **Expanded** | **Contracted** |
| --- | --- | --- |
| Bta | 300 | 60 |
| Cfa | 193 | 113 |
| Bta,Cfa | 254 | 0 |
| Rfe | 141 | 158 |
| Bta,Cfa,Rfe | 273 | 16 |
| Hgl | 124 | 183 |
| Hsa | 166 | 149 |
| Hgl,Hsa | 259 | 4 |
| Hsa,Hgl,Rfe,Bta,Cfa | 232 | 87 |
| Ema | 251 | 59 |
| Laf | 285 | 79 |
| Ema,Laf | 318 | 1 |
| Pca | 90 | 203 |
| Ema,Laf,Pca | 249 | 35 |
| Ete | 110 | 193 |
| Oaf | 181 | 125 |
| Oaf, Ete | 256 | 1 |
| Oaf, Ete, Ema, Laf, Pca | 221 | 47 |
| Oaf,Ete,Ema,Laf,Pca,Hsa,Hgl,Rfe,Bta,Cfa | 260 | 32 |
| Mdo | 124 | 184 |
| Oaf,Ete,Ema,Laf,Pca,Hsa,Hgl,Rfe,Bta,Cfa,Mdo | 248 | 8 |
| Oan | 48 | 240 |

Notes: Bta: *B. taurus*; Cfa: *C. familiaris;* Rfe: *R. ferrumequinum*; Ete: *E. telfairi*; Hgl: *H. glaber*; Hsa: *H. sapiens*; Ema: *E. maximus*; Laf: *L. africiana*; Mdo: *M. domestica*; Oaf: *O. afer*; Oan: *O. anatinu*s; Pca: *P.capensis*.

**Supplementary Table S13.** Gene Ontology (GO) of significantly expanded gene families in the Asian elephant and African bush elephant.

| **ID** | **Class** | ***P*-value** | **Corrected *P-*value** | **Count** | **Description** |
| --- | --- | --- | --- | --- | --- |
| GO:0005085 | MF | 2.80E-13 | 1.04E-11 | 19 | Guanyl-nucleotide exchange factor activity |
| GO:0003924 | MF | 1.93E-09 | 3.56E-08 | 18 | GTPase activity |
| GO:0007264 | BO | 6.82E-07 | 8.41E-06 | 19 | Small GTPase mediated signal transduction |
| GO:0005525 | MF | 1.51E-06 | 1.39E-05 | 20 | GTP binding |

**Supplementary Table S14.** KEGG enrichment analysis of significantly expanded gene families in the ancestral lineages of Asian elephant and African bush elephant.

| **ID** | ***P*-value** | **Corrected *P-*value** | **Count** | **Description** |
| --- | --- | --- | --- | --- |
| ko05310 | 2.74E-22 | 2.52E-20 | 40 | Asthma |
| ko04015 | 8.36E-20 | 3.84E-18 | 66 | Rap1 signaling pathway |
| ko05210 | 2.02E-19 | 4.64E-18 | 37 | Colorectal cancer |
| ko05212 | 2.02E-19 | 4.64E-18 | 37 | Pancreatic cancer |
| ko04072 | 5.39E-19 | 9.91E-18 | 51 | Phospholipase D signaling pathway |
| ko04014 | 1.33E-18 | 2.03E-17 | 65 | Ras signaling pathway |
| ko05322 | 1.62E-14 | 2.13E-13 | 43 | Systemic lupus erythematosus |
| ko05144 | 1.48E-13 | 1.70E-12 | 28 | Malaria |
| ko05332 | 3.57E-13 | 3.65E-12 | 29 | Graft-versus-host disease |
| ko05321 | 3.30E-11 | 3.03E-10 | 27 | Inflammatory bowel disease (IBD) |
| ko05202 | 1.32E-10 | 1.10E-09 | 46 | Transcriptional misregulation in cancer |
| ko04020 | 3.82E-09 | 2.93E-08 | 42 | Calcium signaling pathway |
| ko00053 | 2.01E-08 | 1.32E-07 | 12 | Ascorbate and aldarate metabolism |
| ko00040 | 1.64E-07 | 1.01E-06 | 12 | Pentose and glucuronate interconversions |
| ko04145 | 2.17E-07 | 1.25E-06 | 32 | Phagosome |
| ko00983 | 4.57E-06 | 2.34E-05 | 12 | Drug metabolism - other enzymes |
| ko00860 | 7.80E-06 | 3.77E-05 | 12 | Porphyrin and chlorophyll metabolism |
| ko05143 | 8.19E-06 | 3.77E-05 | 14 | African trypanosomiasis |
| ko05340 | 9.43E-06 | 4.13E-05 | 14 | Primary immunodeficiency |
| ko00982 | 4.89E-05 | 2.04E-04 | 12 | Drug metabolism - cytochrome P450 |
| ko04514 | 5.45E-05 | 2.18E-04 | 28 | Cell adhesion molecules (CAMs) |
| ko00980 | 1.06E-04 | 4.06E-04 | 12 | Metabolism of xenobiotics by cytochrome P450 |
| ko00140 | 1.20E-04 | 4.40E-04 | 12 | Steroid hormone biosynthesis |
| ko00830 | 2.65E-04 | 9.36E-04 | 12 | Retinol metabolism |
| ko05204 | 7.80E-04 | 2.66E-03 | 12 | Chemical carcinogenesis |
| ko04664 | 1.02E-03 | 3.36E-03 | 14 | Fc epsilon RI signaling pathway |
| ko04662 | 1.37E-03 | 4.34E-03 | 14 | B cell receptor signaling pathway |
| ko05140 | 2.52E-03 | 7.71E-03 | 14 | Leishmaniasis |
| ko03008 | 8.56E-03 | 2.54E-02 | 11 | Ribosome biogenesis in eukaryotes |
| ko05166 | 8.86E-03 | 2.55E-02 | 27 | HTLV-I infection |
| ko05134 | 1.09E-02 | 3.05E-02 | 10 | Legionellosis |

**Supplementary Table S15.** Expanded gene families on the ancestral branch of Asian elephant and African bush elephant

| **Gene families** | **Laf** | **Ema** | **Bta** | **Cfa** | **Ete** | **Hgl** | **Hsa** | **Mdo** | **Oan** | **Pca** | **Rfe** | **Oaf** |
| --- | --- | --- | --- | --- | --- | --- | --- | --- | --- | --- | --- | --- |
| ZNF | 5 | 31 | 26 | 20 | 3 | 17 | 101 | 341 | 5 | 6 | 27 | 15 |
| ZNF | 2 | 70 | 0 | 3 | 10 | 2 | 5 | 0 | 0 | 4 | 3 | 24 |
| IGKV | 8 | 11 | 1 | 1 | 1 | 8 | 22 | 21 | 1 | 0 | 15 | 1 |
| EEF1A1 | 11 | 10 | 3 | 19 | 5 | 14 | 1 | 3 | 3 | 1 | 7 | 3 |
| UGT | 16 | 12 | 12 | 5 | 1 | 5 | 9 | 1 | 2 | 2 | 2 | 2 |
| GBP | 9 | 9 | 11 | 2 | 3 | 6 | 7 | 6 | 4 | 4 | 3 | 3 |
| NXF | 12 | 10 | 1 | 5 | 3 | 2 | 3 | 2 | 1 | 2 | 2 | 3 |
| OR | 6 | 4 | 1 | 8 | 2 | 2 | 1 | 1 | 3 | 1 | 2 | 7 |
| OR | 10 | 4 | 4 | 2 | 1 | 5 | 2 | 2 | 0 | 0 | 1 | 6 |
| OR | 4 | 4 | 1 | 4 | 0 | 6 | 3 | 2 | 1 | 1 | 6 | 2 |
| LRRC | 7 | 5 | 1 | 2 | 0 | 0 | 4 | 1 | 1 | 1 | 2 | 3 |
| NUTM | 7 | 5 | 1 | 1 | 1 | 0 | 6 | 0 | 0 | 0 | 2 | 1 |
| TRIM | 12 | 3 | 0 | 0 | 0 | 1 | 1 | 0 | 0 | 0 | 1 | 1 |

**Supplementary Table S16.** Gene Ontology (GO) enrichment of significantly expanded gene families in the Asian elephant.

| **GO** | **Class** | ***P*-value** | **Corrected *P-*value** | **Count** | **Description** |
| --- | --- | --- | --- | --- | --- |
| GO:0007156 | BP | 6.74E-31 | 2.22E-29 | 34 | Hemophilic cell adhesion via plasma membrane adhesion molecules |
| GO:0005886 | CC | 4.72E-28 | 7.79E-27 | 34 | Plasma membrane |
| GO:0007155 | BP | 1.55E-25 | 1.70E-24 | 34 | Cell adhesion |
| GO:0005509 | MF | 6.56E-17 | 5.41E-16 | 42 | Calcium ion binding |
| GO:0046872 | MF | 2.42E-05 | 1.14E-04 | 28 | Metal ion binding |
| GO:0016020 | CC | 1.03E-04 | 3.79E-04 | 36 | Membrane |
| GO:0005622 | CC | 1.36E-02 | 4.48E-02 | 18 | Intracellular |
| GO:0003676 | MF | 3.69E-02 | 1.11E-01 | 16 | Nucleic acid binding |

Notes: The GO terms with corrected *p*-value bellow 0.05 are selected as significant enriched groups.

**Supplementary Table S17.** KEGG enrichment analysis of significantly expanded gene families in the Asian elephant.

| **ID** | ***P*-value** | **Corrected *P-*value** | **Count** | **Description** |
| --- | --- | --- | --- | --- |
| ko05310 | 2.98E-32 | 3.01E-30 | 47 | Asthma |
| ko04015 | 9.14E-32 | 4.62E-30 | 77 | Rap1 signaling pathway |
| ko05322 | 2.62E-30 | 8.82E-29 | 59 | Systemic lupus erythematosus |
| ko04014 | 4.59E-27 | 1.16E-25 | 72 | Ras signaling pathway |
| ko05144 | 5.52E-22 | 1.12E-20 | 35 | Malaria |
| ko04020 | 1.96E-19 | 2.20E-18 | 55 | Calcium signaling pathway |
| ko05332 | 2.34E-19 | 2.36E-18 | 34 | Graft-versus-host disease |
| ko05321 | 7.65E-19 | 7.03E-18 | 34 | Inflammatory bowel disease (IBD) |
| ko05202 | 7.96E-16 | 6.70E-15 | 51 | Transcriptional misregulation in cancer |
| ko04514 | 8.51E-10 | 6.14E-09 | 35 | Cell adhesion molecules (CAMs) |
| ko04664 | 1.02E-06 | 6.04E-06 | 18 | Fc epsilon RI signaling pathway |
| ko04662 | 1.59E-06 | 8.90E-06 | 18 | B cell receptor signaling pathway |
| ko05166 | 3.08E-06 | 1.48E-05 | 34 | HTLV-I infection |
| ko03410 | 6.74E-05 | 3.09E-04 | 11 | Base excision repair |
| ko04214 | 5.18E-04 | 2.27E-03 | 11 | Apoptosis |
| ko04212 | 2.22E-03 | 8.97E-03 | 11 | Longevity regulating pathway |

**Supplementary Table S18.** Expanded gene families on the branch of Asian elephant

| **Gene families** | **Ema** | **Laf** | **Ete** | **Hgl** | **Hsa** | **Mdo** | **Oan** | **Pca** | **Rfe** | **Oaf** | **Cfa** | **Bta** |
| --- | --- | --- | --- | --- | --- | --- | --- | --- | --- | --- | --- | --- |
| PCDH | 34 | 6 | 7 | 9 | 21 | 15 | 18 | 12 | 14 | 29 | 10 | 7 |
| ZNF | 22 | 1 | 10 | 3 | 7 | 0 | 0 | 7 | 6 | 7 | 5 | 5 |
| ZNF | 12 | 2 | 5 | 5 | 11 | 0 | 0 | 8 | 5 | 10 | 7 | 6 |
| ZNF | 8 | 1 | 2 | 5 | 5 | 0 | 0 | 5 | 8 | 5 | 4 | 4 |
| ZNF | 7 | 0 | 1 | 5 | 5 | 0 | 0 | 2 | 4 | 3 | 2 | 3 |
| ZNF | 7 | 2 | 2 | 1 | 3 | 0 | 0 | 2 | 1 | 4 | 2 | 1 |
| ZNF | 7 | 0 | 2 | 0 | 2 | 0 | 0 | 2 | 1 | 4 | 0 | 0 |
| ZNF | 6 | 1 | 1 | 2 | 12 | 0 | 0 | 1 | 3 | 3 | 3 | 3 |
| OR | 5 | 2 | 6 | 1 | 1 | 1 | 0 | 1 | 0 | 2 | 2 | 2 |

**Supplementary Table S19.** Positively selected genes identified in the Asian elephant and African bush elephant

See the materials **Sup_table19_PSGs_group1**.

**Notes**: *p*-value or *q*-value = 0 indicates that <1e^-17^.

**Supplementary Table S20.** KEGG pathway and Gene Ontology (GO) enrichment analysis of positively selected genes identified in the Asian elephant and African bush elephant.

| **Category** | **ID** | **Description** | ***P*-value** | **Count** |
| --- | --- | --- | --- | --- |
| KEGG Pathway | ko05217 | Basal cell carcinoma | 0.01 | 7 |
| KEGG Pathway | ko00730 | Thiamine metabolism | 0.01 | 3 |
| KEGG Pathway | ko03440 | Homologous recombination | 0.01 | 5 |
| KEGG Pathway | ko05169 | Epstein-Barr virus infection | 0.02 | 16 |
| KEGG Pathway | ko04520 | Adherens junction | 0.03 | 8 |
| KEGG Pathway | ko05213 | Endometrial cancer | 0.03 | 6 |
| KEGG Pathway | ko00561 | Glycerolipid metabolism | 0.04 | 5 |
| KEGG Pathway | ko04013 | MAPK signaling pathway - fly | 0.04 | 8 |
| KEGG Pathway | ko03008 | Ribosome biogenesis in eukaryotes | 0.04 | 7 |
| KEGG Pathway | ko00310 | Lysine degradation | 0.05 | 6 |
| GO Molecular Functions | GO:0020037 | Heme binding | 0.001 | 5 |
| GO Molecular Functions | GO:0008536 | Ran GTPase binding | 0.003 | 3 |
| GO Cellular Components | GO:0005871 | Kinesin complex | 0.01 | 3 |
| GO Molecular Functions | GO:0003779 | Actin binding | 0.02 | 4 |
| GO Molecular Functions | GO:0003777 | Microtubule motor activity | 0.02 | 3 |
| GO Molecular Functions | GO:0016705 | Oxidoreductase activity, acting on paired donors, with incorporation or reduction of molecular oxygen | 0.02 | 3 |
| GO Molecular Functions | GO:0005524 | ATP binding | 0.03 | 18 |
| GO Biological Processes | GO:0007018 | Microtubule-based movement | 0.03 | 3 |
| GO Biological Processes | GO:0055114 | Oxidation-reduction process | 0.03 | 9 |
| GO Molecular Functions | GO:0008168 | Methyltransferase activity | 0.03 | 3 |
| GO Molecular Functions | GO:0003743 | Translation initiation factor activity | 0.04 | 2 |
| GO Molecular Functions | GO:0008026 | ATP-dependent helicase activity | 0.04 | 2 |
| GO Molecular Functions | GO:0016746 | Transferase activity, transferring acyl groups | 0.04 | 2 |
| GO Molecular Functions | GO:0008017 | Microtubule binding | 0.04 | 5 |

**Supplementary Table S21.** Positively selected genes identified in the Asian elephant, African bush elephant, naked-mole rat and greater horseshoe bat.

See the materials **Sup_table21_PSGs_group4.**

**Notes**: *p*-value or *q*-value = 0 indicates that <1e^-17^

**Supplementary Table S22.** KEGG pathway and Gene Ontology (GO) enrichment analysis of positively selected genes identified in the Asian elephant, African bush elephant, naked-mole rat and greater horseshoe bat.

| **Category** | **ID** | **Description** | **p-value** | **Count** |
| --- | --- | --- | --- | --- |
| KEGG Pathway | ko05217 | Basal cell carcinoma | 0.01 | 3 |
| KEGG Pathway | ko00514 | Other types of O-glycan biosynthesis | 0.02 | 2 |
| KEGG Pathway | ko04540 | Gap junction | 0.02 | 3 |
| KEGG Pathway | ko04520 | Adherens junction | 0.03 | 3 |
| KEGG Pathway | ko03022 | Basal transcription factors | 0.04 | 2 |
| GO Molecular Functions | GO:0003779 | Actin binding | 0.001 | 3 |
